# Diet composition influences the metabolic benefits of short cycles of very low caloric intake

**DOI:** 10.1038/s41467-021-26654-5

**Published:** 2021-11-09

**Authors:** Alberto Diaz-Ruiz, Tyler Rhinesmith, Laura C. D. Pomatto-Watson, Nathan L. Price, Farzin Eshaghi, Margaux R. Ehrlich, Jacqueline M. Moats, Melissa Carpenter, Annamaria Rudderow, Sebastian Brandhorst, Julie A. Mattison, Miguel A. Aon, Michel Bernier, Valter D. Longo, Rafael de Cabo

**Affiliations:** 1 Experimental Gerontology Section, Translational Gerontology Branch, National Institute on Aging, National Institutes of Health, Baltimore, MD 21224 USA; 2 Nutritional Interventions Group, Precision Nutrition and Aging, Institute IMDEA Food, Crta. de Canto Blanco n° 8, E – 28049 Madrid, Spain; 3 Longevity Institute, School of Gerontology, and Department of Biological Sciences, University of Southern California, Los Angeles, CA 90089 USA; 4 Nonhuman Primate Core, Translational Gerontology Branch, National Institutes of Health, National Institute on Aging, Dickerson, MD 20842 USA; 5 Laboratory of Cardiovascular Science, National Institute on Aging, National Institutes of Health, Baltimore, MD 21224 USA; 6 IFOM, FIRC Institute of Molecular Oncology, 20139 Milano, Italy

**Keywords:** Ageing, Metabolism, Obesity

## Abstract

Diet composition, calories, and fasting times contribute to the maintenance of health. However, the impact of very low-calorie intake (VLCI) achieved with either standard laboratory chow (SD) or a plant-based fasting mimicking diet (FMD) is not fully understood. Here, using middle-aged male mice we show that 5 months of short 4:10 VLCI cycles lead to decreases in both fat and lean mass, accompanied by improved physical performance and glucoregulation, and greater metabolic flexibility independent of diet composition. A long-lasting metabolomic reprograming in serum and liver is observed in mice on VLCI cycles with SD, but not FMD. Further, when challenged with an obesogenic diet, cycles of VLCI do not prevent diet-induced obesity nor do they elicit a long-lasting metabolic memory, despite achieving modest metabolic flexibility. Our results highlight the importance of diet composition in mediating the metabolic benefits of short cycles of VLCI.

## Abstract (web summary)

Understanding the contribution of diet composition, calories and length of fasting in health maintenance is still challenging. Here the authors compare the effects of cycles of intermittent very low calorie intake achieved with a plant-based fasting mimicking diet or standard laboratory chow to provide insights into the role played by diet composition in mediating the metabolic benefits of short cycles of very low-calorie intake in mice.

## Introduction

Sedentary lifestyle and Western dietary habits are among the main contributors to the rise in obesity around the world ^1^; World Health Organization. Obesity and Overweight. WHO; 2020 (https://www.who.int/mediacentre/factsheets/fs311/en/)]. High energy intake, low energy expenditure, and excess weight gain are associated with metabolic disorders, comorbidities (e.g., type 2 diabetes and cardiovascular disease), and overall increase in mortality^2^, whereas intentional weight loss induces favorable effects on health and reduces all-cause mortality^3^. Studies in rodents have documented a wide range of health-related benefits associated with a reduction in calorie intake, including weight loss, improvement of insulin sensitivity, reduction of chronic inflammation, prevention of cardiovascular and neurological diseases, and anti-cancer protection^4–6^. Of note, calorie restriction (CR), time-restricted feeding, and intermittent fasting are nutritional interventions that slow the progression of age-associated symptoms while extending lifespan in animal models^7,8^. Variations in whole-body physiology, reduction of circulating glucose/growth factors, modulation of circadian rhythms, use of ketone bodies, and overall enhancement of metabolic flexibility are among the molecular mechanisms underlying the salutary impact of CR and related nutritional approaches on health and longevity^9–13^. Based on this evidence, intentional fasting has gained increasing interest for optimizing health in normal weight subjects, and to combat metabolic disease in overweight/obese population^4,14–16^.

Dietary strategies have emerged in which daily eating patterns are adjusted either by means of meal size, frequency of feeding and/or length of fasting time, with or without reduction of total energy intake^7,15,17,18^. These interventions comprise periods of low-calorie intake alternating with periods of normal food consumption^15^, and include intermittent fasting (IF), alternate days of energy restriction, and time-restricting feeding (TRF). Recent reports describe the health benefits of 4:10 cycles of severe CR using a plant-based “fasting-mimicking diet (FMD)” for 4 days followed by ad libitum feeding for 10 days^19–22^. However, whether these benefits of FMD are driven by the diet composition, the reduction in calorie intake well below daily needs, the length of fasting or a combination of these is not fully understood. Noteworthy, many of these strategies are often non-sustainable and fraught with potential negative side effects and ills, including difficulty to maintain living activities, experiencing low mood, stress, and sustained hunger, which, in turn, translates into hyperphagia in non-restricted days and alterations of eating behaviors^7,23,24^. Therefore, optimization of nutritional interventions to maintain compliance, reduce side effects, and help individuals achieve metabolic advantages is much needed. In addition, although previous clinical trials in patients on CR and short intermittent bouts of energy restriction have documented equivalent reduction in body weight and cardiometabolic risk [reviewed in refs. ^16,25^], little is known about the long-term benefits of CR vs. intermittent periods of low energy intake, such as the 4:10 feeding protocol, nor the safety of these interventions in overweight/obese subjects^16,26–28^.

In this work, we have implemented a 5-month intervention where middle-aged male mice are exposed to 4:10 feeding cycles of severe CR with standard laboratory chow (AIN-93G, LCC) or a less severe Fasting-Mimicking Diet (FMD) for 4 days every two weeks for 11 cycles. A 10-day ad libitum access to AIN-93G concludes each cycle. Here we show specific changes with respect to whole-body physiology, metabolic and bioenergetic status, as well as the dynamic response to stimuli by metabolite profiling. Notably, the diet composition exerts an important role in mediating the metabolic benefits of short cycles of VLCI.

## Results

### Health improvements caused by cycles of very low-calorie intake (VLCI) in mice are diet dependent

**Fig. 1:**
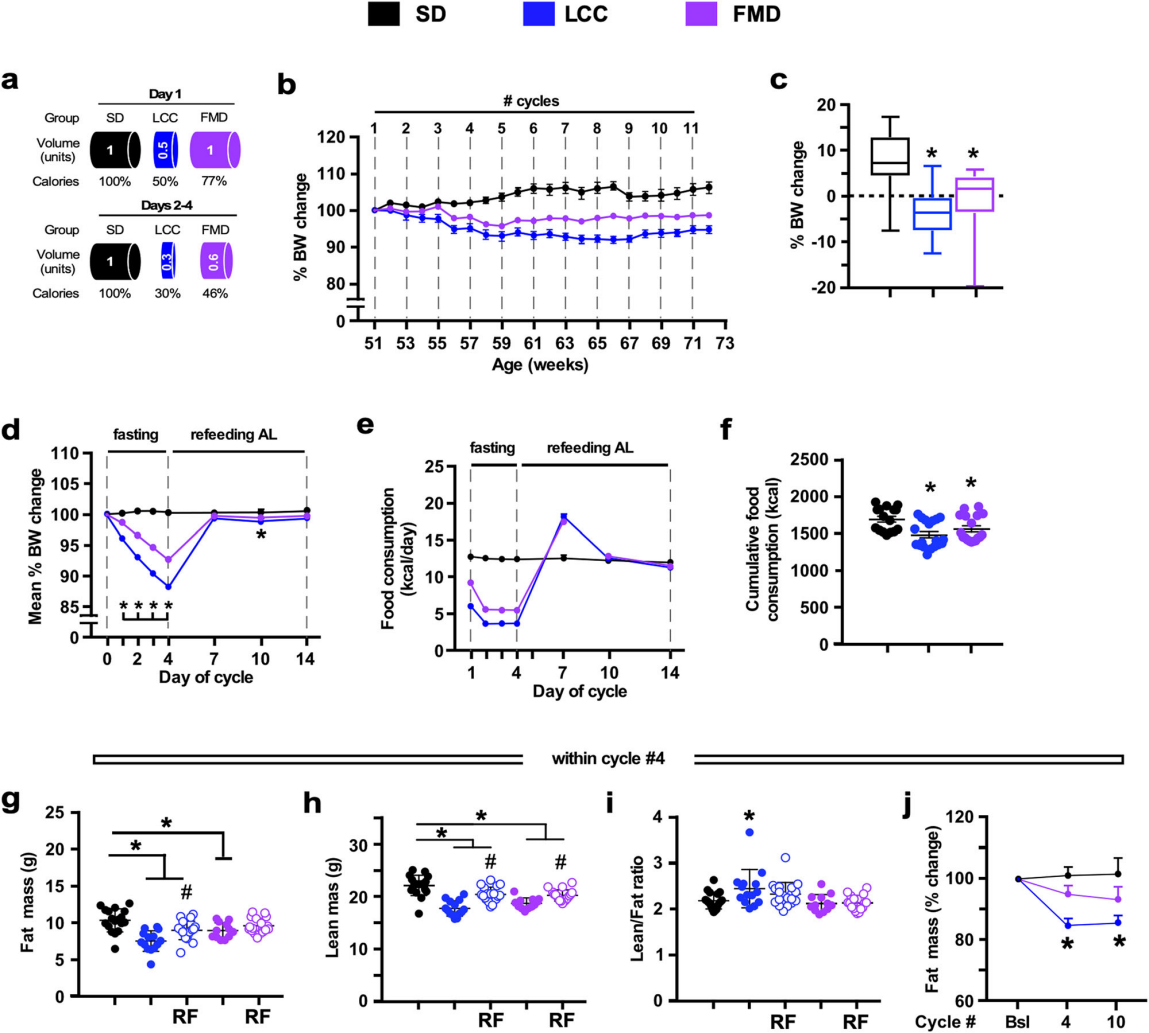
Effect of 4:10 feeding cycles on body weight, feeding behavior, and body composition in mice maintained on SD. **a** Pictorial representation of the feeding regime. SD, standard diet (black symbols); LCC, low-calorie cycles (blue symbols); FMD, fasting-mimicking diet (purple symbols). Following a 4-day period of reduced calorie intake, mice were returned to ad libitum diet (AL) for 10 days before initiating a new round of 4:10 fasting-refeeding cycle for a total of 11 cycles. **b** Body weight trajectories as percent change from baseline (SD, *n* = 16–18; LCC, *n* = 19; FMD, n = 17–18). **c** Cumulative percent body weight changes from baseline to cycle 11 (SD, *n* = 16; LCC, *n* = 19; FMD, *n* = 18). Data are represented as box plot [center line, median; box limits, upper and lower quartiles; whiskers minimum and maximum values]. **d** Average percent body weight change during a representative 4:10 cycle (*n* = 11 cycles). Body weight measurements were taken at days 1–4, 7, 10, and 14 of each cycle; **e** Average food consumption (kCal) on each day of a representative 4:10 cycle (*n* = 11 cycles). **f** Cumulative food consumption (kCal) from baseline to cycle 11 (SD, *n* = 16; LCC, *n* = 19; FMD, *n* = 18); **g** Measures of body fat and (**h**) lean mass by NMR, and (**i**) lean-to-fat ratio calculation during cycle 4. Measures for SD, LCC and FMD groups were collected on day 4, while the measures for LCC-RF and FMD-RF were acquired on day 14. Comparison by one-way ANOVA: *, *p* < 0.05 vs. SD; #, *p* < 0.05 vs. LCC or FMD, respectively (SD, *n* = 18; LCC, *n* = 14; LCC-RF, *n* = 19; FMD, *n* = 12; FMD-RF, *n* = 18); **j** Percent change in fat mass from baseline (Bsl) during the refed phase of cycles 4 and 10, respectively (SD, *n* = 15/15/13; LCC, *n* = 17/17/15; FMD, *n* = 16/16/16). *, *p* < 0.05 vs. SD.

To evaluate the health influence of cycles of VLCI cycles, we conducted a 5-month study where 12-month-old male C57BL/6 mice were maintained on a standard diet (SD) ad libitum (AL) (SD-AL group) or subjected to 4:10 feeding regimen [LCC (Low-Calorie Cycles) and FMD groups]. Within each of the 4-day cycles, LCC mice were given SD but at a 50:70:70:70% reduction in daily calories whereas FMD mice received a diet with a fasting-mimicking composition at a 33:54:54:54% reduction in daily calories (Fig. 1a and Supplementary Fig. 1a). At the end of the 4-day cycle, both LCC and FMD mice returned to AL refeeding (RF) with SD for the next 10 days. SD-AL animals exhibited a ~6.9% weight gain over the 5 months, while LCC and FMD groups lost ~4.3% and ~0.9% weight, respectively (Fig. 1b, c and Supplementary Fig. 1b, c). Mice lost ~8–12% weight during each cycle [LCC~12%; FMD ~8%] and regained most of the weight upon RF (Fig. 1d). Returning to AL feeding caused the LCC and FMD mice to gorge, a likely compensatory behavior for the VLCI cycle (Fig. 1e and Supplementary Fig. 1d). Despite this behavior, a ~12.4% and a ~7.7% reduction in the average of total calorie consumption per mouse over the 5-month study was observed in LCC and FMD mice vs. SD-AL controls, respectively (Fig. 1f). Cumulative calorie intake normalized to body weight remained identical for all groups of mice (Supplementary Fig. 1e). On cycle #4 of the study, the total amount of fat and lean body mass was significantly lower in LCC mice vs. SD-AL controls at the end of the 4-day period (Fig. 1g, h, respectively), resulting in the greater lean-to-fat ratio (Fig. 1i). After 7 days of RF, LCC-RF and FMD-RF mice partially regained fat and lean body mass, with concurrent lower lean-to-fat ratios (Fig. 1g-i). On cycle #10, there was a significant reduction in both fat and lean body mass, expressed as % change from baseline, in LCC mice along with a transient increase in the lean-to-fat ratio at cycle 4 but not at cycle 10 (Fig. 1j and Supplementary Fig. 1f, g). At the end of the 4-day period of low-calorie intake, FMD-fed mice displayed a reduction in fat and lean body mass, which did not result in a significant alteration in the lean/fat ratio (Fig. 1g–i). On the other hand, analysis of fat and lean body mass trajectories from baseline to cycles #4 and #10 indicated that FMD-fed mice had less lean tissue, while the reduction in fat mass was not found to be statistically significant and no changes in the lean-to-fat ratio were observed at either time point (Fig. 1j and Supplementary Fig. 1f, g). These results indicate that the implementation of 4:10 cycles of VLCI with either LCC or FMD is an effective strategy for reducing body weight and total fat mass in the context of SD. However, FMD had a small advantage over LCC in preventing lean body mass loss likely because FMD was less severely restrictive in calories consumed.

**Fig. 2:**
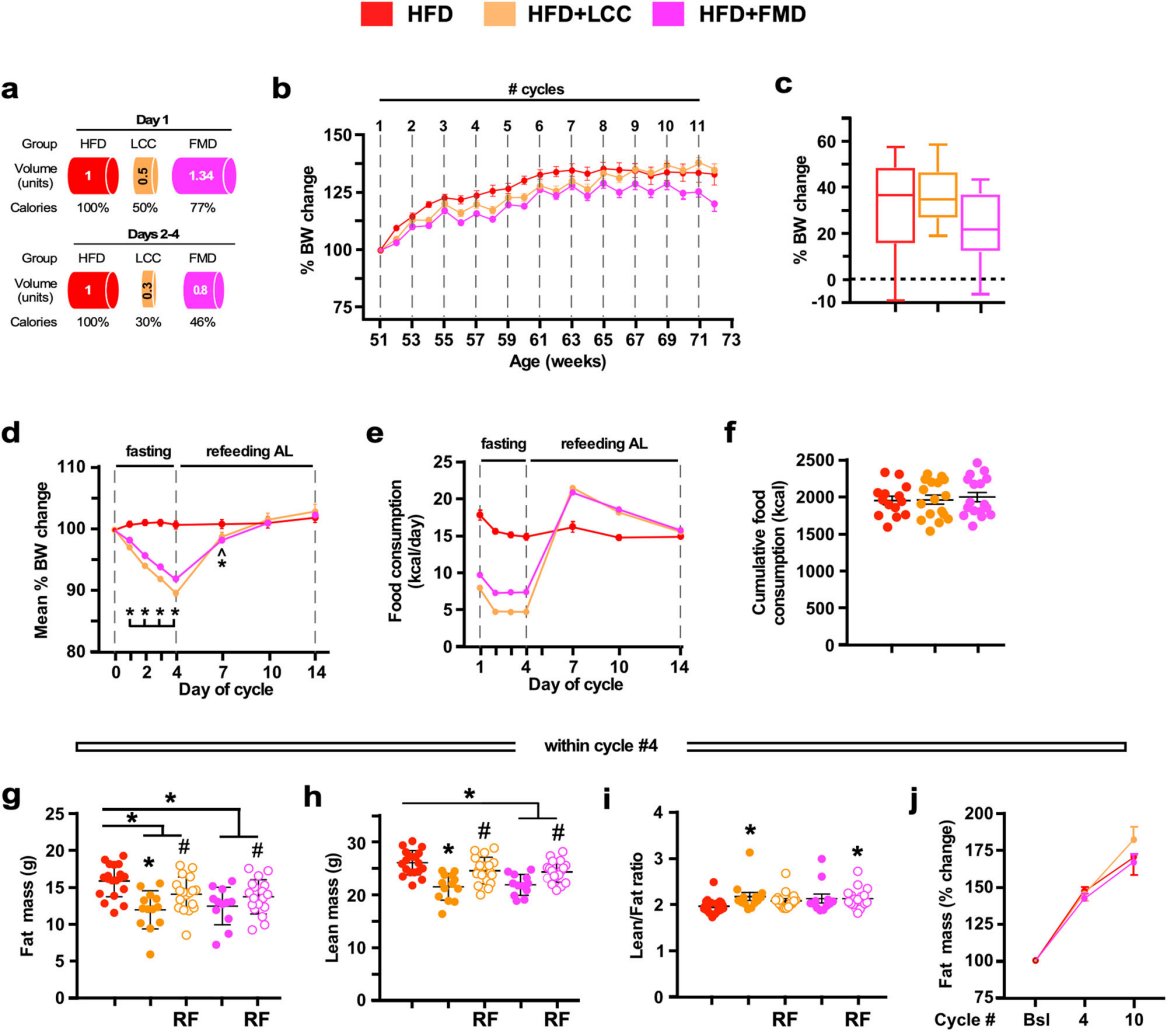
Effect of 4:10 cycles on body weight, feeding behavior, and body composition in mice fed on HFD. **a** Representation of the feeding regime. HFD, high-fat diet (red symbols); HFD + LCC, cycles of low-calorie intake under HFD (orange symbols); HFD + FMD, cycles of fasting-mimicking diet under HFD (pink symbols). Mice underwent a 4-day period of reduced energy restriction followed by a 10-day AL regime before initiating a new round of 4:10 fasting-refeeding cycle for a total of 11 cycles. **b** Body weight trajectories as percent change from baseline (HFD, *n* = 16–19; HFD + LCC and HFD + FMD, *n* = 17–18). **c** Cumulative percent body weight changes from baseline to cycle 11 (HFD, *n* = 16; HFD + LCC and HFD + FMD, *n* = 17). Data are represented as box plot [center line, median; box limits, upper and lower quartiles; whiskers minimum and maximum values]. **d** Average percent body weight change on each day of a representative 4:10 cycle (*n* = 11 cycles); **e** Average food consumption (kCal) on each day of a representative 4:10 cycle (*n* = 11 cycles). **f** Cumulative food consumption (kCal) from baseline to cycle 11 (HFD, *n* = 16; HFD + LCC and HFD + FMD, *n* = 17). **g** Measures of body fat and (**h**) lean mass by NMR and (**i**) lean-to-fat ratio calculation during cycle 4. Measures for HFD, HFD + LCC and HFD + FMD groups were collected on day 4, while the measures for HFD + LCC-RF and HFD + FMD-RF were acquired on day 14. Comparison by one-way ANOVA: *, *p* < 0.05 vs. HFD; #, *p* < 0.05 vs. HFD + LCC or HFD + FMD, respectively (HFD, *n* = 19; HFD + LCC, *n* = 13; HFD + LCC-RF, *n* = 18; HFD + FMD, *n* = 11; HFD + FMD-RF, *n* = 18). **j** Percent change in fat mass from baseline (Bsl) during refed phase of cycles 4 and 10, respectively (HFD, *n* = 17/16/13; HFD + LCC, *n* = 15/15/13; HFD + FMD, *n* = 14/14/11). All data are expressed as mean ± SEM. Comparison by two-tailed t-test unless otherwise noted; ^*p* < 0.1, *p < 0.05.

To study the effectiveness of the 4:10 intervention to prevent dietary-induced obesity, another cohort of mice were fed a high-fat diet (HFD) under AL conditions or subjected to a 4:10 regimen. HFD + LCC and HFD + FMD mice had, respectively, a 50:70:70:70% and 33:54:54:54% reduction of daily intake during the 4-day period before returning to AL-HFD for the next 10 days (Fig. 2a). The efficacy of the 4:10 cycles at reducing body weight gain progressively declined throughout the study, although HFD + FMD mice maintained the body weight loss much better than HFD + LCC animals (Fig. 2b, c and Supplementary Fig. 2a, b). Mice lost ~10% weight during each cycle [HFD + LCC ~11%; HFD + FMD ~9%], regaining most of the weight upon RF (Fig. 2d). Gorging behavior also occurred upon return of AL feeding (Fig. 2e and Supplementary Fig. 2c); however, cumulative food consumed by HFD + LCC and HFD + FMD mice before (Fig. 2f) and after body weight normalization (Supplementary Fig. 2d) was comparable to that of HFD-AL. On cycle #4, the total fat and lean body mass of HFD + LCC and HFD + FMD mice were significantly reduced vs. HFD-AL controls at the end of the 4-day period (Fig. 2g, h), with the higher lean-to-fat ratio in HFD + LCC mice (Fig. 2i). Upon RF, there was an incomplete return of fat and lean body mass to HFD-AL values, with higher lean-to-fat ratio in HFD + FMD-RF mice (Fig. 2g-i). At cycles #4 and #10, the three groups of mice had similar trajectories of body fat mass, expressed as % change from baseline (Fig. 2j), while HFD + LCC and HFD + FMD mice exhibited a significant reduction in lean mass along with lower lean-to-fat ratio (Supplementary Fig. 2e, f). Lean mass did return to baseline in HFD + LCC-RF mice, but not in HFD + FMD-RF mice. Overall, these findings indicate that implementation of cycles of VLCI in the context of an obesogenic diet failed to maintain body weight loss and reduction in total fat mass. Cumulative food consumption was comparable among the three experimental groups (Fig. 2f), and yet HFD + FMD mice could maintain body weight loss better than HFD + LCC mice even though the 4-day low-calorie intake with FMD was much less restrictive than with LCC diet (Fig. 2e). We can only surmise the emergence of marked changes in energy efficiency (∆ body weight change/kCal ingested) between the two groups, with LCC diet providing better substrates for growth.

**Fig. 3:**
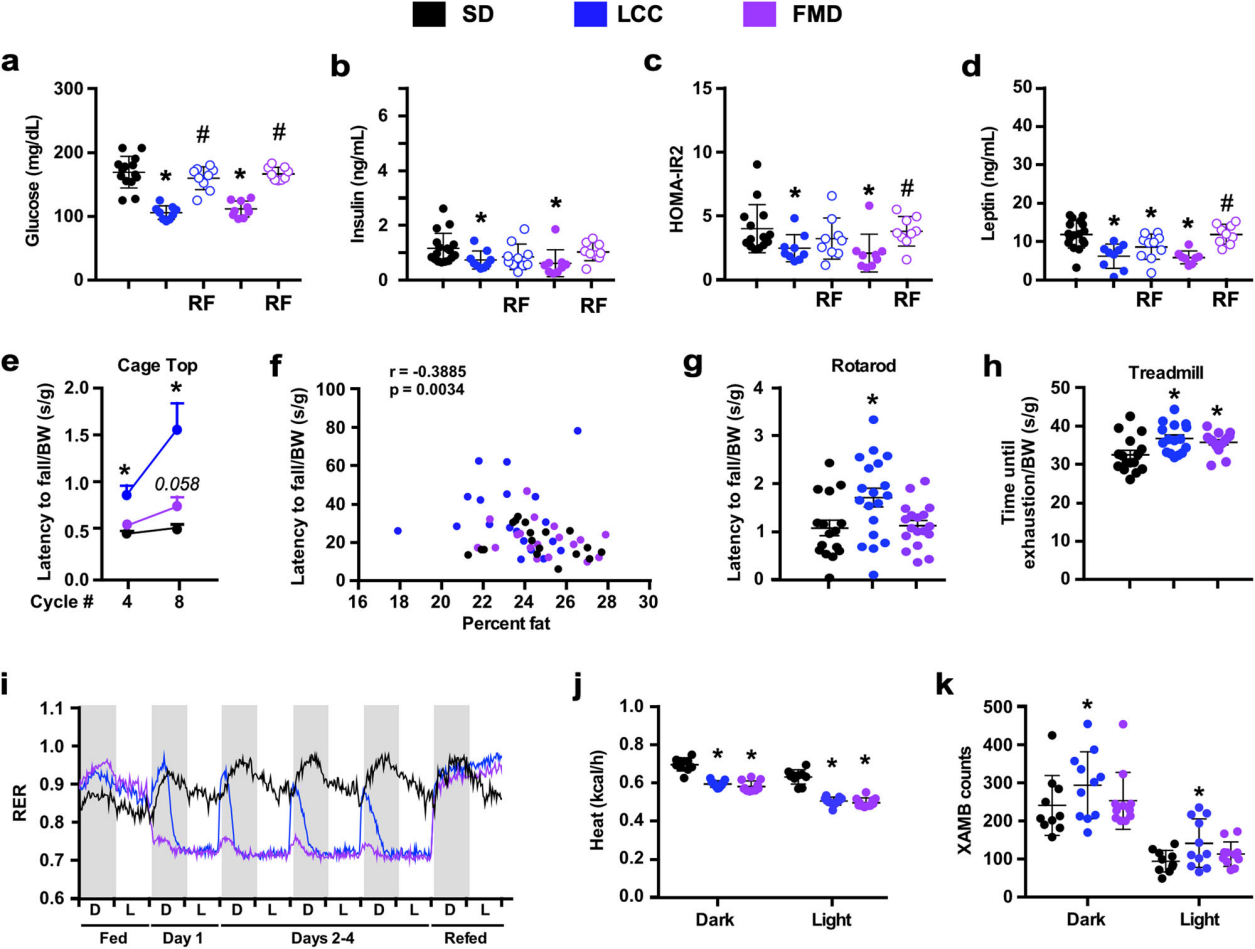
Effect of 4:10 feeding cycles on health biomarkers in mice on SD. **a**–**d** Measures of circulating glucose, insulin, HOMA-IR2 calculation, and leptin levels during cycle 9. Serum from SD was collected on day 4 and day 11. Serum from LCC and FMD was collected on day 4, while serum from LCC-RF and FMD-RF was collected on day 11. Comparison by one-way ANOVA: *, *p* < 0.05 vs. SD; #, *p* < 0.05 vs. LCC or FMD, respectively (SD, *n* = 14-17; LCC, *n* = 9; LCC-RF, *n* = 9–10; FMD, *n* = 8–9; FMD-RF, *n* = 9). **e**–**h** All physical performance values were expressed as latency to fall (seconds) normalized by body weight (BW) in grams. **e** Inverted cage top t**e**sts were conducted on days 10–13 of cycles 4 and 8 (SD, *n* = 18/17; LCC, *n* = 19/19; FMD, *n* = 18/18); **f** Spearman’s correlation between latency to fall from cage top and percent body fat during cycle 4. **g** Rotarod tests were conducted on day 11 of cycle 8 (SD, *n* = 16; LCC, *n* = 19; FMD, *n* = 18). **h** Treadmill endurance test during the refeeding period of cycle 4. Data were collected from an independent cohort of mice (*n* = 16) at the age of 26 weeks. See “Methods” for detailed information. **i**–**k** Mice fed SD were placed into metabolic cages during cycles 3 and 4 to measure (**i**) the respiratory exchange ratio (RER) (SD, *n* = 7–10; LCC and FMD, *n* = 8–11 per group), (**j**) averaged hourly heat production, and (**k**) ambulatory activity counts (SD, *n* = 10; LCC and FMD, *n* = 11 per group). Dark/light cycle represented in panel (**i**) by alternate gray/wh**i**te bars.

We next analyzed several circulating biomarkers of metabolic health. In the context of SD, glucose, insulin, and leptin levels and HOMA-IR2 (homeostatic measure of insulin resistance) index were significantly reduced at the end of the VLCI period in LCC and FMD mice (Fig. 3a–d). In LCC-RF mice (after 7 days of RF), leptin levels remained significantly lower, a pattern consistent with the downward trend in glucose and insulin levels, whereas the FMD-RF group had these values returning close to SD-AL controls (Fig. 3a–d). Subjecting mice to 4:10 feeding cycles did not alter glucose disposal during glucose tolerance tests (OGTTs) (Supplementary Fig. 3a). Physical performance was then investigated by assessing traditional inverted cage top, rotarod, and treadmill tests while the mice were on days 10–14 of the 4:10 cycle (refed phase). The latency to fall values (in seconds) or distance traveled were subjected to normalization by body weight (grams), lean body mass (grams) or represented as absolute values. Regardless of data normalization, LCC mice outperformed FMD mice in these physical tests overall, as denoted by the fact that improved performance on cage top test was observed for LCC mice at 2 and 4 months while the response in FMD mice at 4 months was less substantial and did not reach statistical significance (*p* = 0.06) vs. SD-AL controls (Fig. 3e and Supplementary Fig. 3c, f). The use of the momentum of impact energy (in Newtons) is a more accurate determination for the latency to fall from the cage top, and the results once again showed better outcome in LCC mice (Supplementary Fig. 3i). Latency to fall from the cage top negatively correlated with percent body fat (Fig. 3f) but it exhibited a positive association with the lean-to-fat ratio (Supplementary Fig. 1h). Mice on LCC, but not FMD, had greater coordination and balance than SD-AL mice, as measured by the rotarod test after 4 months of intervention, with and without normalization by body weight or lean body mass (Fig. 3g and Supplementary Fig. 3d, g). However, both groups of mice performed significantly better on the treadmill than SD-AL controls without and with normalization by body weight (Fig. 3h and Supplementary Fig. 3e). Overall, we show that 4:10 feeding cycles increased physiological and metabolic fitness, characterized by weight loss, improved glucose homeostasis and insulin sensitivity, as well as the reduction in body fat and lean muscle mass, all leading to enhanced physical performance, especially in LCC mice.

Variations in energy expenditure elicited by cycles of VLCI were assessed on cycles #3 and #4 of the study by placing mice in metabolic cages over a 6-day period (1 day fed—4 days on VLCI—1 day RF). Compared to SD-AL controls, LCC mice exhibited larger respiratory exchange ratio (RER) amplitudes—ranging from ~0.70 to ~0.95 during the day/fasting to night/feeding cycle, respectively, indicating enhanced metabolic flexibility (Fig. 3i). FMD mice consumed their food at a much slower rate (Supplementary Fig. 3b) and had very small postprandial amplitudes during the same 4-day period (Fig. 3i), likely reflecting the impact that diet composition has on the type of substrate utilization (CHO vs. fats). One day of ad libitum RF caused RER values to sharply rise, indicating greater utilization of CHO as a fuel source (Fig. 3i). Significant reduction in heat production (Fig. 3j) and a trend toward increased ambulatory activity (Fig. 3k and Supplementary Fig. 3j) were observed under VLCI, thus recapitulating key features of energy restriction^29^. We investigated the known association between energy expenditure (EE) and body weight and found that middle-aged SD-AL and LCC mice were only showing poor correlations in relation to body weight and energy metabolism (Supplementary Fig. 3k). This is consistent with our recent work demonstrating that the normally close association between body weight and respiratory parameters is diminished in middle-aged and old animals^30^.

**Fig. 4:**
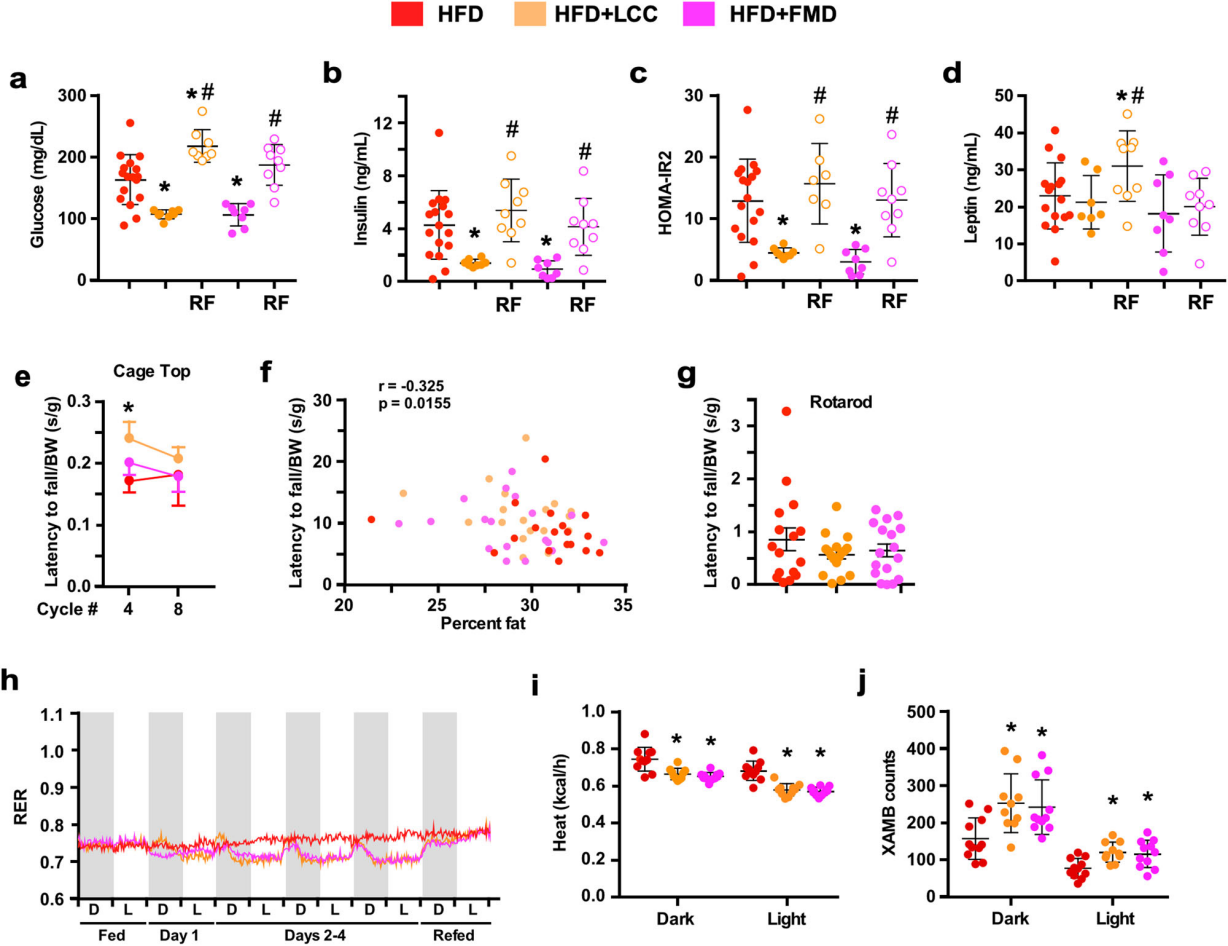
Effect of 4:10 feeding cycles on health biomarkers in mice maintained on HFD. **a**–**d** Circulating glucose, insulin, HOMA-IR2 calculation, and leptin levels in HFD-fed mice during cycle 9. Serum from HFD was collected on day 4 and day 11; Serum from HFD + LCC and HFD + FMD was collected on day 4; while serum from HFD + LCC-RF and HFD + FMD-RF was collected on day 11; Comparison by one-way ANOVA: *, *p* < 0.05 vs. HFD; #, *p* < 0.05 vs. LCC or FMD, respectively (HFD, *n* = 17; HFD + LCC, *n* = 8; HFD + LCC-RF, *n* = 7–9; HFD + FMD, *n* = 8; HFD + FMD-RF, *n* = 9). **e**–**g** All physical performance values were expressed as latency to fall (seconds) normalized by body weight (BW) in grams. **e** Inverted cage top t**e**sts were conducted on days 10–13 of cycles 4 and 8. (HFD, *n* = 18/17; HFD + LCC, *n* = 19/19; HFD + FMD, *n* = 18/17). **f** Spearman’s correlation between latency to fall from cage top and percent body fat during cycle 4. **g** Rotarod tests were conducted on day 11 of cycle 8 (HFD, *n* = 16; HFD + LCC and HFD + FMD, *n* = 17 per group). **h**–**j** Mice fed HFD were placed into metabolic cages during cycles 3 and 4 to measure **h** RER (HFD, *n* = 8–11; HFD + LCC, *n* = 8–10; HFD + FMD, *n* = 9–11), (**i**) averaged hourly heat production, and (**j**) ambulatory activity counts, (HFD, *n* = 11; HFD + LCC, *n* = 10; HFD + FMD, *n* = 11). Dark/light cycle represented in panel (**h**) by alternate gray/white bars. All data are expressed as mean ± SEM. Comparison by two-tailed t-test unless otherwise noted; *p < 0.05 vs. SD or HFD controls.

At the end of the 4-day period of VLCI, HFD + LCC and HFD + FMD mice had reduced fasting blood glucose and plasma insulin levels with significantly lower HOMA-IR2 index than HFD-AL controls (Fig. 4a–c). Unchanged plasma leptin levels were found between the three groups of mice (Fig. 4d). These parameters returned to HFD-AL levels after 7 days of RF, except for a surge in glucose and leptin in HFD + LCC mice (Fig. 4a–d). HFD + LCC and HFD + FMD mice subjected to 4:10 cycles did not alter glucose disposal during OGTTs (Supplementary Fig. 4a). On cycle #4, HFD + LCC mice showed better performance in inverted cage top tests vs. HFD-AL controls after the latency to fall values were normalized by body weight or lean mass but not in absolute terms (Fig. 4e and Supplementary Fig. 4d vs. b). The cage top results negatively correlated with percent body fat (Fig. 4f) but did not exhibit any association with the lean-to-fat ratio (Supplementary Fig. 2g). No group differences were found in the rotarod tests after 4 months of dietary intervention with or without body weight normalization (Fig. 4g and Supplementary Fig. 4e).

As anticipated, mice on HFD displayed a clear metabolic shift towards FA utilization accompanied by reduced circadian rhythmicity in fuel utilization (Fig. 4h). HFD + LCC and HFD + FMD mice relied greatly on FA oxidation during the 4-day period (RER ~ 0.7), with shorter postprandial peaks in both amplitude and duration. RER values were comparable between the three groups of mice, both before and after the food reduction cycle. Very low-calorie diet associated with a significant reduction in heat generation (Fig. 4i) and enhanced spontaneous locomotor activity (Fig. 4j and Supplementary Fig. 4f). Contrary to HFD-AL controls and HFD + LCC mice, the reduction in EE in HFD + FMD mice was not influenced by body weight (as a covariate). The increased range of body weights in these animals likely contributes to the improved correlation between body weight and energy expenditure compared to middle-aged mice fed a standard diet. Consistent with this, the FMD group, where the increased body weight is less pronounced, did not show a strong correlation (Supplementary Fig. 4g). Altogether, these results provide insight into differences in the way LCC and FMD modulate energy metabolism.

### Serum and hepatic metabolomic responses to feeding paradigm and diet composition

Potential metabolic signatures associated with the salutary effects of 4:10 feeding cycles were investigated. At the end of the 5-month intervention (cycle #11), mice were sacrificed in the morning on day 3 (‘VLCI’ state) or day 10 (6 days of RF). VLCI mice were fed the previous afternoon during a 2-h window (between 3:30 and 5:30 pm), whereas RF mice were fed AL.

**Fig. 5:**
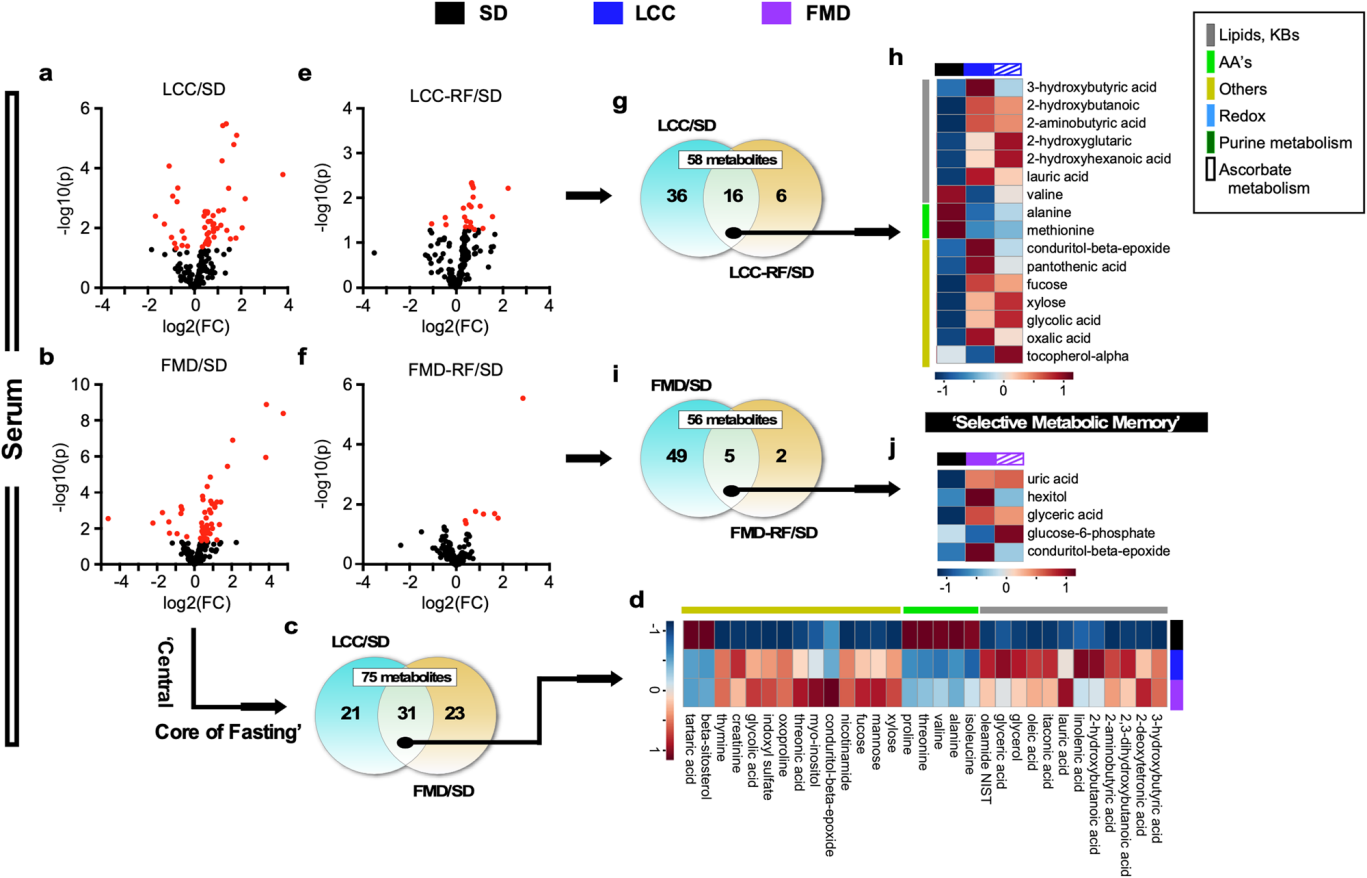
Long-lasting metabolic signature of severe calorie restriction in serum of LCC mice upon refeeding. Untargeted serum metabolomics was carried out in LCC and FMD vs. SD-AL mice. Serum from the SD-AL group was collected on day 3 and 11 (*n* = 7), serum from severely calorie-restricted LCC and FMD mice on day 3 (*n* = 6–7), and serum from refed (RF) LCC and FMD animals on day 10 (*n* = 4) of the last 4:10 cycle. **a**, **b** Volcano plots of metabolites contributing to the separation between a LCC and b FMD mice after severe CR vs. SD-AL controls, respectively. **c**, **d** Venn diagram and heatmap illustrate the core metabolites in serum, representing metabolites associated with a very low-calorie intake that were shared between **c** LCC and FMD mice and **d** their relative average signal vs. SD-AL controls. **e**, **f** Volcano plot of metabolites contributing to the separation between (**e**) LCC-RF and (**f**) FMD-RF mice vs. SD-AL controls, respectively. **g**, **h** Venn diagram and heatmap illustrate a metabolic signature given by metabolites that were preserved between (**a**) the severely calorie-restricted LCC and (**e**) LCC-RF groups and (**h**) their relative average signal vs. SD-AL controls. Red asterisks indicate those metabolites that are part of the metabolic signature depicted in panel (**d**). **i**, **j** Venn diagram and heatmap of preserved metabolites between (**b**) the severely calorie-restricted FMD and (**f**) FMD-RF mice and (**j**) their relative average signal vs. SD-AL controls. See Supplementary Table 1 for a complete list of significantly impacted metabolites by LCC and FMD 4:10 cycles in serum (**a**, **b**, **e**, **f**). See Supplementary Tables 2, 3 for signature metabolites after severe CR (**d**, serum) and their persistence after RF (**h**, serum), respectively.

Untargeted mass spectrometry metabolomics was used first to compare serum and liver profiles of LCC and FMD mice versus SD-fed animals, and significantly changed metabolites (fold-change up ≥ 1.2 or down ≤ 0.83, p < 0.05) were visualized using volcano plots. A subset of 52 and 54 fully annotated metabolites (out of 194) were found to be significantly altered in the serum of LCC and FMD mice on VLCI for 3 days (Fig. 5a, b and Supplementary Table 1). Of these, 31 metabolites were common between the LCC:SD and FMD:SD pairwise comparisons (Fig. 5c and Supplementary Table 2) with their relative abundance displayed as a heatmap (Fig. 5d, see Supplementary Fig. 5a for individual mice). The majority of these 31 circulating metabolites included lipids, ketone bodies and amino acids (AAs) in VLCI compared to SD-fed controls. The increase in the ketone body, beta-hydroxybutyrate (3-HB), and lipid species was consistent with enhanced lipolysis in adipose tissue and fat oxidation, whereas the depletion in AAs coincided with lean mass loss (Supplementary Fig. 1f).

A set of 22 circulating metabolites was significantly impacted in LCC mice following a 6-day refeeding period (LCC-RF) compared to SD-AL controls (Fig. 5e). The overall abundance of lipids and 3-HB combined with AA depletion (Supplementary Table 1) was reminiscent to the pattern observed in LCC mice under severe calorie restriction. Unexpectedly, 16 out of 22 metabolites (~67%) were shared between LCC-RF and LCC mice (Fig. 5g, h, Supplementary Fig. 5b, c, and Supplementary Table 3), suggesting the persistence of a metabolic fingerprint of low energy intake despite the return to AL feeding for 6 days. In contrast to the LCC-RF group, the serum metabolome of FMD-RF mice returned only 7 metabolites vs. SD-AL controls (Fig. 5f and Supplementary Table 1), of which 5 were shared with FMD mice (Fig. 5i, j, Supplementary Fig. 5d, and Supplementary Table 3).

**Fig. 6:**
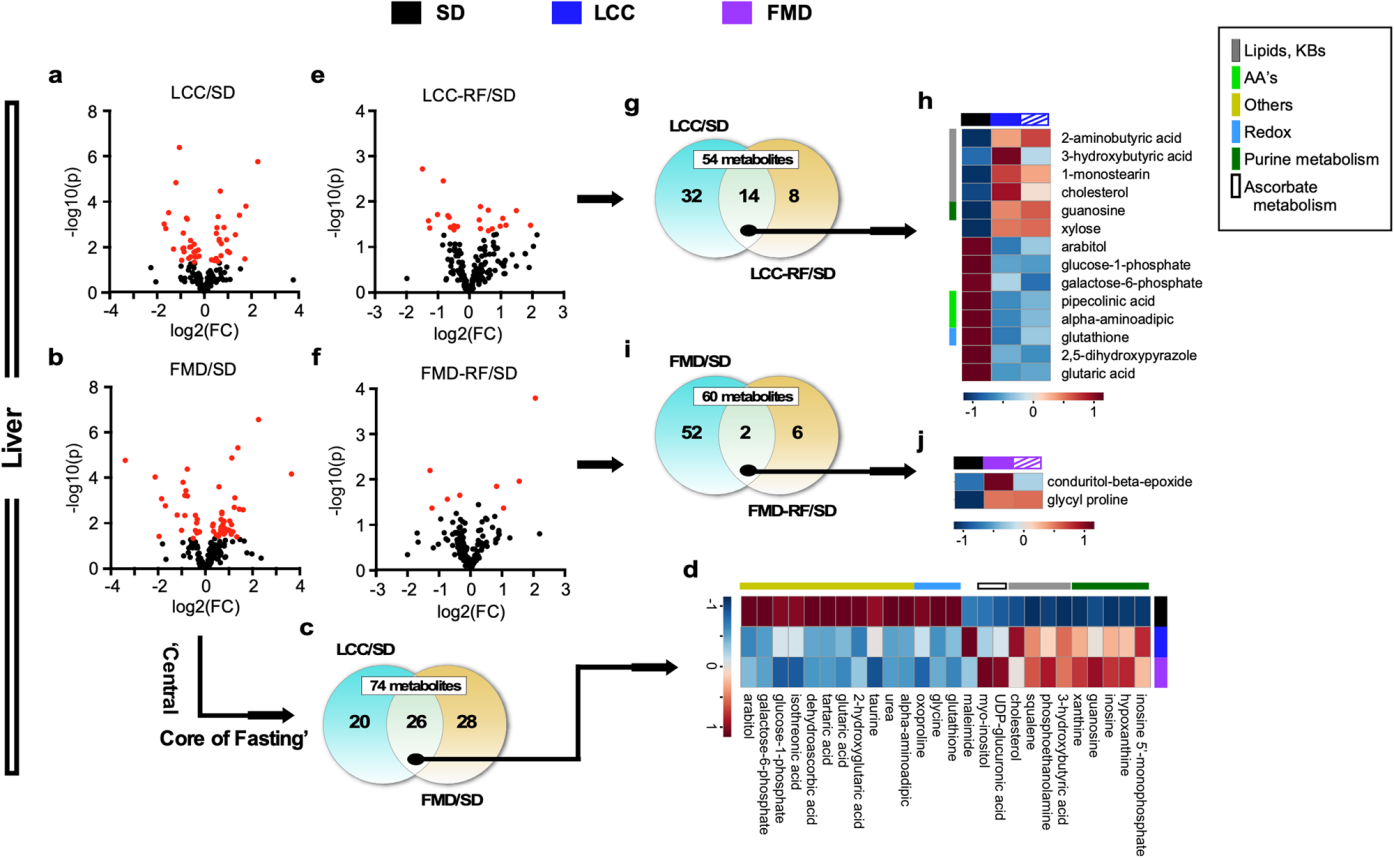
Liver untargeted metabolomics study in LCC and FMD vs. SD-AL mice. Livers from SD-AL controls were collected on day 3 and 11 (*n* = 8), livers from severely calorie-restricted LCC and FMD mice on day 3 (*n* = 7) and those from LCC-RF and FMD-RF animals on day 10 (*n* = 4) of the 4:10 cycle. **a**, **b** Volcano plots showing metabolites accounting for the effect of very low-calorie intake in (**a**) LCC and (**b**) FMD vs. SD-AL livers, respectively. **c**, **d** Venn diagram and heatmap illustrate the core metabolites, representing (**c**) metabolites associated with severe CR that were shared in LCC and FMD livers and (**d**) their relative average signal vs. SD-AL controls. **e**, **f** Volcano plot representing the separation between (**e**) LCC-RF and (**f**) FMD-RF livers vs. SD controls, respectively. **g**, **h** Venn diagram and heatmap illustrate the ‘Selective Metabolic Memory’—e.g., metabolites that were preserved between (**a**) the severely calorie-restricted LCC and (**e**) LCC-RF livers and (**h**) their relative average signal vs. SD-AL controls. **i**, **j** Venn diagram and heatmap of preserved metabolites between (**b**) the severely calorie-restricted FMD and (**f**) FMD-RF livers and (**j**) their relative average signal vs. SD-AL controls. For all volcano plots, red symbols indicate metabolites above threshold (fold-change ≥ 1.2 or ≤ 0.83, raw *p* value ≤ 0.05). See Supplementary Table 1 for a complete list of significantly impacted metabolites by LCC and FMD 4:10 cycles in liver (**a**, **b**, **e**, **f**). See Supplementary Tables 2, 3 for signature metabolites after severe CR (**d**, liver) and their persistence after RF (**h**, liver), respectively.

Using a similar approach to serum, we identified a subset of 46 and 54 metabolites in liver that were significantly altered in the LCC:SD and FMD:SD pairwise comparisons, respectively (Fig. 6a–c and Supplementary Table 1), of which 26 were common (Fig. 6c, d, Supplementary Fig. 5e, and Supplementary Table 2). Pathway analysis with MetaboAnalyst 4.0^31^ unveiled four main top-ranked metabolic descriptors (log *p* > 2.5), e.g., purine, lipids-ketone bodies, ascorbate, and redox (Supplementary Table 4 and Supplementary Fig. 6a, b). Increases in hepatic purine and ascorbate metabolism were evident by the accumulation of several metabolites (e.g., inosine monophosphate, hypoxanthine, xanthine, and uric acid, as well as UDP-glucuronic, hexuronic, and dehydroascorbic, respectively).

The LCC-RF liver showed alteration of 22 metabolites compared to SD-AL controls (Fig. 6e and Supplementary Table 1), of which 14 were shared with LCC mice (Fig. 6g, h, and Supplementary Fig. 5f and Supplementary Table 3), revealing a metabolic fingerprint characterized by up-modulation of lipids-ketone bodies and purine metabolism with concomitant decrease in metabolites associated with lysine degradation and redox pathways (Fig. 6h and Supplementary Table 3). This signature was not observed in FMD-RF liver, which showed alterations in 8 metabolites vs. SD controls (Fig. 6f and Supplementary Table 1), of which only conduritol-β-epoxide and glycylproline were shared with FMD (Fig. 6i, j, Supplementary Fig. 5g, and Supplementary Table 3). Overall, lipid-ketone body accumulation and AA depletion dominated both the liver and serum metabolome of LCC-RF mice, whereas the FMD-RF group showed a much weaker signature reminiscent of the SD-AL controls.

**Fig. 7:**
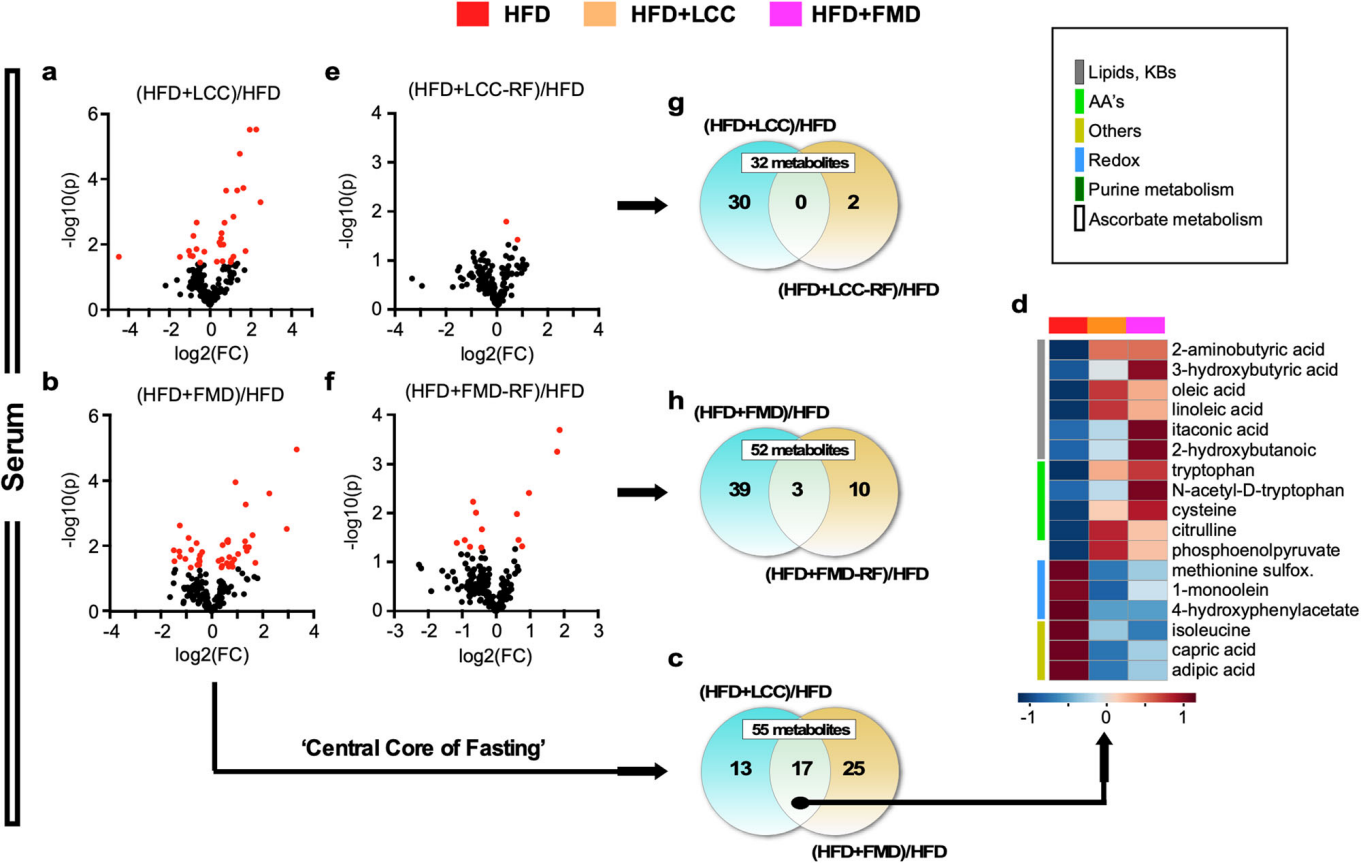
Mice fed an obesogenic diet do not preserve the serum metabolic signature of severe CR after refeeding. **a**–**d** Untargeted serum metabolomics was carried out in LCC and FMD mice fed on HFD and compared to HFD-AL controls. Serum from HFD-AL mice was collected on days 3 and 11 (*n* = 7), serum from severely calorie-restricted HFD + LCC and HFD + FMD mice on day 3 (*n* = 5–6), and serum from refed (RF) HFD + LCC and HFD + FMD animals on day 10 (*n* = 4) of the last 4:10 cycle. **a**, **b** Volcano plots of metabolites contributing to the separation between (**a**) HFD + LCC and (**b**) HFD + FMD mice after severe CR vs. HFD-AL controls, respectively. **c**, **d** Venn diagram and heatmap illustrate the core metabolites in serum, representing those associated with very low-calorie intake that were shared between (**c**) HFD + LCC and HFD + FMD mice and (**d**) their relative average signal vs. HFD-AL controls. **e**, **f** Volcano plot of metabolites contributing to the separation between (**e**) HFD + LCC and (**f**) HFD + FMD mice after refeeding vs. HFD-AL controls, respectively. **g**, **h** Venn diagrams of shared serum metabolites between HFD + LCC or HFD + FMD mice and their refed counterparts [HFD + LCC-RF or HFD + FMD-RF] vs. HFD-AL controls.

Next, we sought to identify metabolic signatures of LCC and FMD in HFD-fed mice and found alterations in 30 and 42 circulating metabolites, respectively, compared to HFD-AL controls (Fig. 7a–c, Supplementary Fig. 7b, c, and Supplementary Table 1). Heatmaps of the 17 shared metabolites showed significant perturbations in descriptors related to lipids-ketone bodies, AAs and redox, all belonging to the central metabolic core of pathways present in HFD + LCC and HFD + FMD mice under VLCI (Fig. 7c, d, Supplementary Fig. 7a, and Supplementary Table 2). The sustained ketogenesis and body weight loss in HFD + FMD mice were accompanied by elevated 3-HB in serum and may reflect the fact that FMD is a plant-based diet. When subjected to refeeding, HFD + LCC-RF mice showed only 2 circulating metabolites (uric acid and glycine) that were significantly altered vs. HFD controls (Fig. 7e and Supplementary Table 1), of which neither was shared with HFD + LCC (Fig. 7g). The serum of HFD + FMD-RF mice yielded 13 metabolites compared to HFD controls (Fig. 7f and Supplementary Table 1), of which 3 were shared with HFD + FMD (Fig. 7h, Supplementary Fig. 7c, red asterisks, and Supplementary Table 3). Thus, it appears that the organismal response to AL refeeding eliminated the VLCI signature in the serum metabolome of HFD-fed mice.

**Fig. 8:**
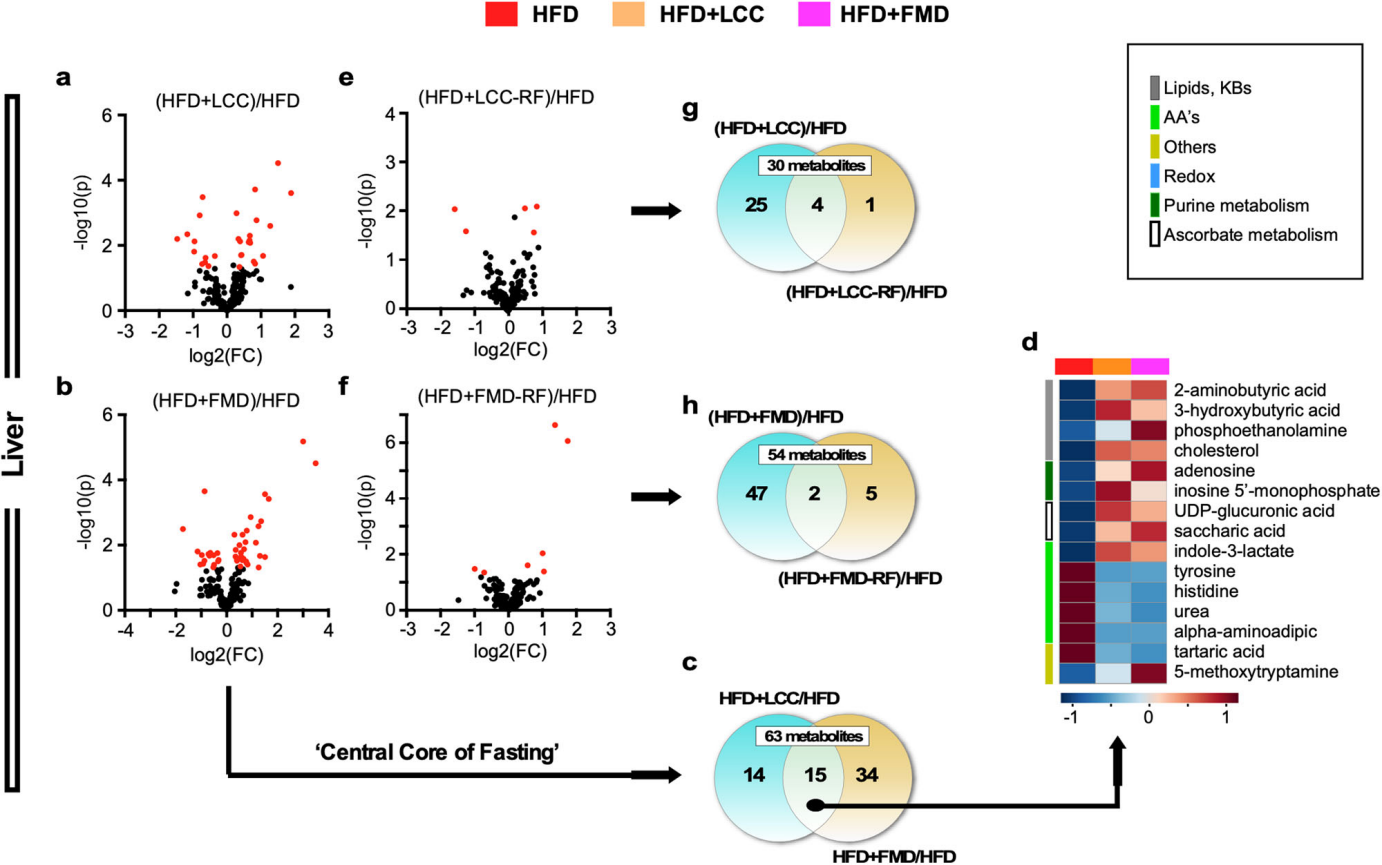
Liver untargeted metabolomics in HFD + LCC and HFD + FMD mice compared to HFD-AL controls. Livers from HFD-AL mice were collected on day 3 and 11 (*n* = 7), livers from severely calorie-restricted HFD + LCC and HFD + FMD mice on day 3 (*n* = 6) and those from HFD + LCC-RF and HFD + FMD-RF animals on day 10 (*n* = 4) of the 4:10 cycle. **a**, **b** Volcano plots show metabolites accounting for the effect of severe CR in (**a**) HFD + LCC and (**b**) HFD + FMD livers vs. HFD-AL controls, respectively. **c**, **d** Venn diagram and heatmap illustrate the core metabolites in liver associated with very low-calorie intake that were shared between (**c**) HFD + LCC and HFD + FMD mice) and (**d**) their relative average signal vs. HFD-AL controls. **e**, **f** Volcano plot representing the separation (**e**) between HFD + LCC and (**f**) HFD + FMD livers after refeeding vs. HFD-AL controls, respectively. **g**, **h** Venn diagrams depicting shared metabolites between (**g**) HFD + LCC or (**h**) HFD + FMD group and their refed counterparts [HFD + LCC-RF or HFD + FMD-RF] vs. HFD-AL controls. For all volcano plots, red symbols indicate metabolites above threshold (fold-change ≥ 1.2 or ≤ 0.83, raw *p* value ≤ 0.05).

Subsets of 29 and 49 metabolites were significantly altered in the liver of calorie-restricted HFD + LCC and HFD + FMD mice vs. HFD controls (Fig. 8a-c, Supplementary Fig. 7e, f, and Supplementary Table 1), of which 15 were common (Fig. 8c, d, Supplementary Fig. 7d, and Supplementary Table 2), revealing a “core” metabolic signature of VLCI that comprised descriptors such as ‘synthesis and degradation of ketone bodies’, ‘ascorbate’ and ‘short-chain fatty acids (butyric)’ as top up-regulated pathways, and AA biosynthesis as the dominant down-regulated pathway (Supplementary Table 4). This VLCI-generated metabolite signature in liver of HFD-fed mice was reminiscent of that in SD-fed animals and was largely mirrored by changes in the serum metabolite profiles. A low degree of overlap in liver metabolites between severely calorie-restricted and refed animals was observed, as HFD + LCC-RF yielded only five metabolites vs. HFD controls (Fig. 8e and Supplementary Table 1) of which 4 were shared with HFD + LCC (Fig. 8g, Supplementary Fig. 7e, red asterisks, and Supplementary Table 3). Of the 7 metabolites significantly impacted in the HFD + FMD-RF_HFD pairwise comparison (Fig. 8h and Supplementary Table 1), only conduritol-β-epoxide and hexitol were common with HFD + FMD (Supplementary Fig. 7f, red asterisks, and Supplementary Table 3).

## Discussion

In this study, middle-aged male mice underwent 4:10 cycles of very-low-calorie intake (VLCI) with two diets differing in composition (e.g., standard laboratory chow (LCC) vs. FMD), in the absence or presence of a high-fat diet (HFD). Mice were assessed first for functional differences associated with physiological and physical performance, followed by an analysis of metabolic remodeling in serum and liver. The results indicate that: (i) 4:10 feeding cycles of SD led to improved physical performance, physiological and metabolic fitness and these changes were independent of the feeding regime (LCC or FMD)"; (ii) repeated cycles of VLCI with standard chow or FMD generated a metabolic memory of fasting in the serum and liver, but not with HFD or HFD + FMD; (iii) untargeted metabolomics of serum and liver at the end of the 4 days of VLCI unveiled a “core” metabolic signature in the liver, shared by LCC and FMD groups, comprising four up-modulated pathways - purines, lipids/ketone bodies, ascorbate, and redox (i.e., glutathione, taurine-hypotaurine); (iv) this metabolic signature was, to a great extent, preserved only with LCC but not FMD, after 6 days of ad libitum refeeding. It involved up-modulation of lipids/ketone bodies and purines metabolism, accompanied by down-regulation of lysine degradation and redox metabolism.

From a physiological perspective, the results showed that HFD + FMD vs HFD + LCC enabled better keeping of body weight loss (Fig. 2b, c). However, LCC outperformed FMD in physical tests overall, despite a more severe calorie restriction, although it remains to be seen whether LCC vs FMD mice isocalorically fed still display superior physiological and metabolic fitness. Mice under 4:10/SD cycles exhibited significantly lower body weight, loss of fat and lean mass, and enhanced physical performance, as evidenced by improved outcomes in muscle strength, motor coordination and balance tests. Muscle mass reduction due to weight loss improves physical performance by decreasing intramuscular fat without adversely affecting muscle strength^32^. Previous studies have shown that the adaptive metabolic responses to dietary switching between regular chow and high-fat diets result in a significant increase in fat deposition and adverse health outcomes^33–35^. In contrast, we found that long-term maintenance of the 4:10/HFD feeding regimen led to weight cycling in the absence of weight loss in the HFD + LCC mice, combined with a sustained reduction of muscle mass and lean-to-fat ratio. Contrary to reports showing that diet-induced body weight fluctuations promote obesity and/or metabolic dysfunction^34,36^, weight cycling was found to be associated with extended longevity in mice as compared to sustained obesity^37^. Differences in sex, strain, age at onset, length of the intervention, and degree of energy restriction could have contributed to these divergent results.

Cycles of FMD decrease circulating levels of insulin-like growth factor 1^22^, which may represent a potential mechanism for the sustained body weight reduction and loss in total body fat in HFD + FMD mice, and likely health improvements as suggested by the inverse relationship between performance and whole-body fat percentage (Fig. 3f, 4f)^19^. Benefits in insulin sensitivity and glucose homeostasis constitute physiological hallmarks of fasting-based nutritional interventions. Both insulinopenia and hypoleptinemia are required for proper organismal control of glucose homeostasis during starvation^38,39^. Under 4:10/SD regimen, severely calorie-restricted LCC and FMD mice exhibited low circulating glucose levels, insulin, leptin, and lower values for HOMA-IR2 compared to AL-fed controls. The fact that mice undergoing 4:10/HFD regimen had ‘normal’ leptin levels during the 4 days of calorie restriction suggests a sustained obesity-associated leptin-resistance^40^ that may negatively influence glycemic control.

Untargeted liver and serum metabolomics during the 4:10 cycles of VLCI enabled us to delineate the metabolic remodeling underlying the physiological and physical performance of mice subjected to different feeding regimes (LCC or FMD) with and without HFD. Mice on 4 days of VLCI exhibited a marked decrease in RER despite distinct fast-fed transitions. The robust and sustained increase in lipid and ketone body levels in both serum and liver was consistent with low (~0.7) RER values during 4 days of severe CR under LCC or FMD. The postprandial swings in RER with large vs. negligible amplitudes between LCC vs. FMD, respectively, underscored clear differences in metabolic flexibility and fuel utilization for energy needs. Our finding of elevated levels of lipids in both serum and liver of LCC-RF mice after 6 days of refeeding is consistent with a lasting metabolic memory induced by LCC. These effects may be partly driven by white adipose tissue (WAT) that, as recently proposed, could represent a nutritional memory source in mediating lifespan extension in response to dietary restriction^41^. Interestingly, sustained hypoleptinemia observed in LCC-RF mice could be responsible for their metabolic signature, since leptin modulates the glucose-fatty acid cycle^38^.

Liver ascorbate metabolism ranked at the top of the ‘central core of VLCI’ regardless of feeding regime (LCC or FMD) or HFD consumption. Ascorbic acid (vitamin C) is a reducing agent and antioxidant that acts as a cofactor in reactions catalyzed by Cu^+^-dependent monooxygenases and Fe^2+^-dependent dioxygenases. These two classes of enzymes are involved in a wide range of cellular processes such as carnitine biosynthesis, fatty acid metabolism, and purine hydroxylation leading to the formation of uric acid^42,43^. In mice, ascorbate is synthesized from D-glucuronate, which is formed through direct enzymatic hydrolysis of uridine diphosphate-glucuronate^43,44^. Intermediates from this pathway, including UDP-glucuronic, hexuronic, and dehydroascorbic acids, were present in the hepatic metabolome of LCC and FMD mice.

VLCI intake elicited a metabolic phenotype characterized by a hepatic pattern of sustained lipid enrichment (short-chain fatty acids propionic, butyric, 2-hydroxybutyric) and ketone bodies (3-hydroxybutyric) along with a general depletion of AAs from the circulation. These findings recapitulate the metabolic remodeling response to severe CR, consisting of a hepatic shift in substrate selection from glucose to fatty acids and AAs, leading to the production of ketone bodies and activation of the urea cycle, respectively^45,46^. This evolutionarily conserved adaptive mechanism has been shown to be responsible for many of the beneficial effects associated with energy restriction^46,47^. The VLCI-elicited reduction in serum AA levels, including branched-chain amino acids (BCAAs), is consistent with higher hepatic uptake of AAs for gluconeogenesis in LCC and FMD mice. Mice fed a diet low in BCAAs have improved metabolic health and, conversely, elevated BCAAs in serum predict impaired insulin signaling and the development of T2D in humans^48^. Here, serum BCAAs (isoleucine and valine) were significantly reduced under VLCI and returned to control levels upon AL refeeding. Thus, the relative depletion in circulating BCAAs may contribute to the health-associated benefits of the 4:10/SD regimen^49^. HFD-fed mice subjected to VLCI under LCC and FMD also had high levels of serum AAs, consistent with protein breakdown associated with leptin and insulin resistance^48^. Under low-caloric conditions, the liver of HFD-fed mice relies heavily on AA utilization for gluconeogenesis and ketone body generation rather than being reused for protein biosynthesis.

Metabolites from de novo, salvage, and degradation purine pathways were consistently expressed in the liver from mice fed with LCC or FMD during low-calorie intake, with a weaker impact under the HFD regime. Purines participate not only in nucleic acid synthesis, but they also play a central role in energy metabolism and contribute to autocrine and paracrine signaling through activation of purinergic receptors^50^. The significant accumulation of purine metabolites in LCC and FMD mice is indicative of active purine degradation pathway^51^ and suggests an imbalance between the salvage and de novo pathways, which, in turn, could lead to the impaired provision of nucleotides to proliferating cancer cells^52^, and reentry of quiescent stem cells into the proliferative cycle^53^.

Together, the findings reported herein provide experimental clues into the potential benefits that can be gained from very low-calorie feeding cycles and add to our understanding of the physiological and metabolic mechanisms that underlie the enhanced cellular and organismal health observed under these conditions. The metabolic remodeling promoted by VLCI induces a shared core of pathways between LCC and FMD, in the presence or absence of HFD, comprising pathways from ascorbate, purines, lipids-ketone bodies, and redox metabolism. A selective metabolic ‘footprint’ signature that partially recapitulates the “core” pathways, as represented by lipids and ketone bodies, persists in LCC mice even after several days of AL refeeding following VLCI.

### Study limitations

Even though differential calorie intake limits a direct comparison between LCC and FMD, independent validation of the biochemical pathways associated with the sustained metabolomic signature of LCC after AL refeeding is required. Variables that influence the response to very low energy intake, such as sex, strain, age of onset, and degrees and length of VLCI, remain to be tested. Lastly, longitudinal studies in mice of both sexes are needed to fully assess the long-term impact of 4:10 feeding cycles with varied diet compositions on health markers, physical performance, aging phenotypes, and lifespan as performed in the SLAM study^54^ with the addition of measurements of resilience.

## Methods

### Animals and husbandry

C57BL/6J male mice were procured from the Jackson Laboratory (Bar Harbor, ME) at the age of 47 weeks. An independent cohort of C57BL/6J male mice was purchased at the age of 10 weeks for the analysis of time-to-meal completion and treadmill test at the age of 20 and 26 weeks, respectively. Mice were single housed in duplexes (Single Housed Duplexed Cage; Dimensions 22.2 × 30.8 × 16.24 cm; Thoren Caging Systems, Hazeltown, PA) upon arrival with autoclaved corncob bedding and a nestlet for enrichment at the NIA Biomedical Research Center (Baltimore, MD). Low-velocity HEPA filtered air was supplied through sealed shelf plenums directly into the cage through air supply orifices above the cage filter top. All mice were acclimated for at least 1 month to Standard Diet (SD) [AIN-93G Purified Rodent Diet; Dyets Inc., #110700, Bethlehem, PA] from arrival. Thereafter, mice (*n* = 18–19/group) were randomly assigned to their dietary intervention as specified below and fed either a standard diet (SD), a high-fat diet (HFD) [High Fat AIN-93G Purified Rodent Diet with 60% fat-derived calories from Dyets Inc., #101920, Bethlehem, PA], or the human Fasting-Mimicking Diet (FMD). Detailed description of SD and HFD composition is shown in Supplementary Table 5. The human fasting-mimicking diet (FMD) is a patented plant-based diet kindly provided by Dr. Valter D. Longo, with carbohydrates, fats and proteins accounting for 29, 65.3 and 5.46% calories. The formulation was combined with glycerol and hydrogel (Clear H_2_O) to obtain a final caloric content of 2.9 kcal/g and to achieve binding allowing the formation of pellets for daily food allotments.

Throughout the study, Baltimore City tap water treated by reverse osmosis and then hyperchlorinated to 2-3 ppm was provided ad libitum in individual water bottles for each duplexed cage. Cages were changed on a weekly basis and always prior to the 4-day period of severe calorie restriction to avoid feeding on residual chow and coprophagy. Cages were changed within a biological safety cabinet or change station, with spot changes as needed. Animal rooms were maintained at 22.2 ± 1 °C and 30-70% humidity. The lights were turned off at 6:00 PM and back on at 6:00 AM each day. Animals were inspected twice daily for health issues and veterinary care was provided as needed. During the study, 1 mouse was found dead and 6 were euthanized due to dermatitis (2 mice), abdominal mass (1 mouse), eye mass (1 mouse), neck mass (1 mouse) and head tilt (1 mouse). The criteria for euthanasia were based on an independent assessment by a veterinarian according to AAALAC guidelines. An additional mouse died during NMR. All these mice were removed for the analysis of the data. Animal protocols were approved by the Animal Care and Use Committee (352-TGB-2019) of the National Institute on Aging, National Institutes of Health, and in compliance with all relevant ethical regulations regarding the care and use of research animals.

### Dietary intervention

After at least one month of acclimation to SD, mice were randomly assigned to 6 experimental groups. Control groups of mice were fed ad libitum (AL) to SD and HFD whereas experimental groups underwent 4:10 feeding cycles. Specifically, at the onset of each cycle, LCC and FMD mice were respectively fed either an SD or FMD diet for 4 days at 50:70:70:70% or 33:54:54:54% reduction of daily calories before returning to SD-AL feeding for the following 10 days. Likewise, HFD + LCC and HFD + FMD groups were fed an HFD or FMD diet during the first 4-day period of each cycle at 50:70:70:70% and 33:54:54:54% reduction in daily calories, followed by their return to HFD-AL feeding for the next 10 days. At the end of each 14-day cycle, the feeding protocol was repeated. Food consumption and body weight were recorded on days 1, 2, 3, 4, 5, 8, and 11 of each cycle, and food allotments for LCC, FMD, HFD + LCC and HFD + FMD groups were adjusted accordingly. Mice on reduced calorie intake (VLCI) were fed in the afternoon during a 2-h window between 3:30 and 5:30 pm.

After eleven dietary cycles, mice were killed by cervical dislocation: AL-fed SD and HFD mice were sacrificed while on their respective diets, whereas LCC, FMD, HFD + LCC and HFD + FMD mice were killed on either day 3 (VLCI) or day 10 (refed) of the cycle. All sacrifices were performed in the morning. Tissues were removed and snap-frozen in liquid nitrogen. Whole blood collected during sacrifice was centrifuged to separate serum. Tissues and serum were stored at −80 °C prior to analysis.

### Body composition

Lean, fat, and fluid mass in live mice were measured by nuclear magnetic resonance (NMR) using the Minispec LF90 (Bruker Optics). Measurements were acquired at baseline and at dietary cycles #4 and #10 (see Supplementary Fig. 1a for timeline).

### Indirect respiration calorimetry

In-vivo mouse metabolic rate was assessed by indirect calorimetry in open-circuit Oxymax chambers with CLAMS (Columbus Instruments). In brief, mice were housed singly with water and food on day 14 (refed phase) of dietary cycles #3 (*n* = 5-6/group) and #4 (n = 5-6/group). LCC, FMD, HFD + LCC and HFD + FMD mice received their daily allotment of food during the 4 days of VLCI and then were refed AL on day 5 for another 24 h for a total recording period of 144 h, with the exception of 2–3 mice/group on cycle #3 that only completed 111 h of recording. All mice were acclimatized to monitoring cages for at least 3–6 h before recording began. Over the 6-day period, all mice were maintained at 24 °C under a 12:12-h light-dark cycle (light period 06:00-18:00). Sample air was passed through an oxygen (O_2_) sensor for determination of O_2_ consumption, which was determined by measuring its concentration in air entering the chamber compared with air leaving the chamber. The sensor was calibrated against a standard gas mix containing defined quantities of O_2_, carbon dioxide (CO_2_), and nitrogen (N_2_). Constant airflow (0.6 L/min) was drawn through the chamber and monitored by a mass-sensitive flow meter. The concentrations of O_2_ and CO_2_ were monitored at the inlet and outlet of the sealed chambers for 30 s at 30-min intervals. Ambulatory locomotor activity (both horizontal and vertical) of mice was also monitored with beams that software transforms into counts of beam breaks per unit of time. Data recorded from both cycles were combined for the analysis.

### Time to meal completion

Time-to-meal-completion during days 1-4 of dietary cycle #2 was measured for LCC and FMD mice (*n* = 16 mice). Mice were fed their usual daily allotment of food at the regular time and monitored continuously for 3 h or until they consumed their meal as evidenced by the absence of food pellet remaining in the cage. Data are represented as an average of the number of hours each mouse took to consume its meal.

### Circulating factors and HOMA calculation

Mice were bled by cheek puncture on day-4 and day-10 of the dietary cycles #3 and #9 (see Supplementary Fig. 1a for timeline). All mice were fasted for 6 h (from 7:00 AM-1:00 PM) prior to blood collection. Fasting blood glucose was measured in fresh whole blood using a handheld instant glucose meter Bayer Breeze2 (Bayer, Mishawaka, IN). Additional blood was collected in 1.1 ml Z-Gel tubes (Sarstedt, Nümbrecht, Germany) and centrifuged 15 min at 4 °C at 18,500 × g to separate serum, which was stored at −80 °C. Serum insulin was measured using a mouse ultra-sensitive enzyme-linked immunosorbent assay (Catalog #90080; Crystal Chem, Downers Grove, IL). Circulating levels of 3-hydroxybutyrate (3-HB) and leptin were determined with commercially available kits according to the manufacturer’s instructions [3-HB: Catalog #700190; Cayman Chemicals, Ann Arbor, MI; leptin—Catalog #EZML—82 K; Millipore, Burlington, MA). Insulin resistance was calculated using the HOMA2 Calculator software available from the Oxford Centre for Diabetes, Endocrinology and Metabolism, Diabetes Trials Unit website (http://www.dtu.ox.ac.uk/). *n* = 7–17 per group.

### Physical activity measures and behavioral response

Physical performance was measured by cage top, rotarod, and treadmill tests at various points throughout the study as detailed in Supplementary Fig. 1a. All animals were on days 10–14 of the 4:10 cycle (refed phase) during testing. Cage top was performed by suspending mice upside-down from wire cage hoppers, one meter above a padded cushion. Wire hoppers were held in place and leveled using a custom-built acrylic support structure. Mice were allowed to grip with all four paws and move freely about the hopper, but gentle manual adjustment was used to discourage them from wrapping their tails around the wire. The time from inversion to when the mouse lost its grip was recorded in seconds. Mice rested for 30 min between three consecutive trials, which were used to calculate average latency to fall. For the rotarod test, the time to fall from an accelerating rotarod (4–40 rpm over 5 min; Med Associates Inc., Fairfax, VT) was measured with a maximum trial length of 5 min. The average latency to fall from 3 consecutive trials was computed, with 30 min rest between trials. For the treadmill test, mice were given a habituation trial on day 1 where they were placed on the moving treadmill belt **(**Columbus Instruments, Columbus, OH) at a constant speed of 4 m min^−1^ for 5 min. The following day, each mouse started at 9 m min^−1^ for minutes 0–2, then the treadmill speed was ramped up to 10 m min^−1^ for minutes 2–6, 12 m min^−1^ for minutes 6–10, 14 m min^−1^ for minutes 10–20, 16 m min^−1^ for minutes 20–30, 18 m min^−1^ for minutes 30–40, 20 m min^−1^ for minutes 40–50, 22 m min^−1^ for minutes 50–55, and 23 m min^−1^ for minutes 55-60. Mice were enticed to run by delivering a mild electrical shock by a shock grid located at the back of the treadmill. Three rapid successive electric shocks were the criterion for ending the test. The time ran until exhaustion was recorded and reported in seconds.

### Metabolomics

Untargeted metabolomics analysis on liver extracts and serum obtained at the time of sacrifice was performed at the UC Davis West Coast Metabolomics Center. A description of the metabolomics experiment and associated analysis is provided as Supplementary materials.

### Quantitation and statistical analysis

No statistical methods were used to predetermine sample size. The experiments were not randomized, and investigators were not blinded to allocation during experiments and outcome assessments. All data are expressed as mean ± SEM. Differences between groups were tested by either two-tailed Student’s *t* test using Excel 2019 (Microsoft Corp., Redmond, WA, USA) or one-way ANOVA with uncorrected Fisher’s LSD test using GraphPad Prism v. 6 (GraphPad Software, Inc., La Jolla, CA). Significant correlations were assessed using bivariate Spearman correlation methods. ANCOVA analysis was performed for the analysis of EE vs. body weight changes in all groups of animals. In all cases, *p* < 0.05 was considered statistically significant.

### Reporting summary

Further information on research design is available in the Nature Research Reporting Summary linked to this article.

## Supplementary information

SUPPLEMENTARY INFORMATION

Reporting Summary

## Data Availability

The mass spectrometry metabolomic data generated in this study have been deposited in the Metabolomics Work Bench database under accession code PR001143 (10.21228/M8ZH7D)^55^. All other processed data generated in this study are provided in the Supplementary Information. Source data are provided with this paper.

## Source data

Source Data
