# Supplementary material for: Diet composition influences the metabolic benefits of short cycles of very low caloric intake
Source: Nat Commun. 2021 Nov 9;12:6463. doi: 10.1038/s41467-021-26654-5 (PMC8578605; doi:10.1038/s41467-021-26654-5)
Supplement: Supplementary file 1 — SUPPLEMENTARY INFORMATION [file 41467_2021_26654_MOESM1_ESM.pdf]

Supplementary Information

Diet Composition Determines the Metabolic Benefits of Short Cycles of Very Low Caloric Intake

Alberto Diaz-Ruiz, Tyler Rhinesmith, Laura C.D. Pomatto-Watson, Nathan L. Price, Farzin Eshaghi, Margaux R. Ehrlich, Jacqueline M. Moats, Melissa Carpenter, Annamaria Rudderow, Sebastian Brandhorst, Julie A. Mattison, Miguel A. Aon, Michel Bernier, Valter D. Longo and Rafael de Cabo

INDEX

Methods - Description of metabolomics experiment and associated analysis
Supplementary Fig. 1. Impact of 4:10 feeding cycles on maintained on HFD
SupplementaryFig. 2. Impact of 4:10 feeding cycles on physiological measurements in mice maintained on HFD
Supplementary Fig. 3. Impact of 4:10 feeding cycles on metabolic and biochemical readouts in mice fed SD
Supplementary Fig. 4. Impact of 4:10 feeding cycles on metabolic and biochemical readouts in mice maintained on HFD
Supplementary Fig. 5. Heatmaps of metabolite profiles in serum and liver from mice on SD
Supplementary Fig. 6. Relative quantitation of metabolites related to purine metabolism in liver from mice on SD and HFD
Supplementary Fig. 7. Heatmaps of metabolite profiles in serum and liver from mice on HFD
Supplementary Table 1. List of serum and liver metabolites significantly impacted by LCC and FMD in mice fed SD and HFD
Supplementary Table 2. Metabolites implicated in the 'Central Core of Fasting' in the serum and liver of mice fed SD and HFD
Supplementary Table 3. Metabolites implicated in the 'Metabolic Memory of Fasting' in the serum and liver of mice fed SD and HFD
Supplementary Table 4. Pathway analysis of shared metabolites in the liver of fasted and refed mice under the indicated experimental conditions
Supplementary Table 5. Composition of the SD and HFD diets.

Methods

Description of metabolomics experiment and associated analysis

As described in Petr et al. 2021 (30), tissue and serum were extracted in an acetonitrile:isopropanol:water (3:3:2) solution, vortexed, centrifuged, and the supernatants aliquoted for downstream analysis. After a series of evaporation and reconstitution steps in 50% acetonitrile, internal standards (C08-C30, fatty acid methyl esters) were added to the dried sample, which was then derivatized for trimethylsilylation of acidic protons. Data were acquired using the method as described by Fiehn, 2008 (55) and summed up by Mitchell et al., 2016 (46). In brief, metabolites were measured using a rtx5Sil-MS column (made of 95% dimethyl, 5% diphenyl-polysiloxane coated on fused silica; Restek Corporation; Bellefonte PA) protected by an empty guard column. This chromatography method yields excellent retention and separation of primary metabolite classes (amino acids, hydroxyl acids, carbohydrates, sugar acids, sterols, aromatics, nucleosides, amines, and miscellaneous compounds) with arrow peak widths of 2–3 s and very good within-series retention time reproducibility of better than 0.2 s absolute deviation of retention times. The mobile phase consisted of helium, with a flow rate of 1 mL/min, and injection volume of 0.5mL. The following mass spectrometry parameters were used: a Leco Pegasus IV mass spectrometer with unit mass resolution at 17 spectra s-1 from 80 to 500 Da at -70 eV for elution of metabolites. As a quality control, for each sequence of sample extractions, one blank negative control was performed by applying the total procedure (e.g., all materials and plastic ware) without biological sample. Result files were transformed by calculating the sum intensities of all structurally identified compounds for each sample, and subsequently dividing all data associated with a sample by the corresponding metabolite sum. The resulting data were multiplied by a constant factor to obtain values without decimal places. Intensities of identified metabolites with more than one peak (e.g., for the syn- and anti-forms of methoximated reducing sugars) were summed to only one value in the transformed data set. The original non-transformed data set was retained. Relative metabolite levels represent the MS peak amplitude normalized with respect to the total metabolites returned, but disregarding unknowns that might potentially comprise artifact peaks or chemical contaminants.

In liver and serum 194 metabolites were detected/identified and subsequently analyzed using MetaboAnalyst versions 3.0 and 4.0 (31, 56), utilizing univariate (ANOVA), clustering (heatmaps), and multivariate (partial least square discriminant, PLSD) built-in analytical methods from modules of this web-based platform, as specified. The autoscaling function of MetaboAnalyst used to normalize the metabolomics data closely resembles the Z ratio expression and requires the detection and removal of outliers. Significantly changed metabolites were determined from the ratio LCC/AL or refeeding/AL both in SD or FMD using as threshold (fold-change up ≥ 1.2 or down ≤ 0.83, raw p value < 0.05). The same procedure was utilized in HFD diet.

Significantly up- or down-modulated metabolites were represented as Volcano plots. Shared and unique metabolites among dietary interventions were determined according to Venn diagrams. The groups of significantly modified metabolites in liver, shared and unique, were analyzed with the "Pathways Analysis" module of MetaboAnalyst. Metabolic pathways were considered significantly enriched at log $p > 2.5$, and accordingly ranked. When necessary, univariate analysis of individual metabolites was performed using one-way ANOVA with uncorrected Fisher's LSD comparison test.

REFERENCES

55. Fiehn, O. Extending the breadth of metabolite profiling by gas chromatography coupled to mass spectrometry. Trends Analyt. Chem. 27, 261–269 (2008).

56. Xia, J. & Wishart, D. S. Using MetaboAnalyst 3.0 for comprehensive metabolomics data analysis. Curr. Protoc. Bioinformatics 55, 14.10.1-14.10.91 (2016).

Figure Legends

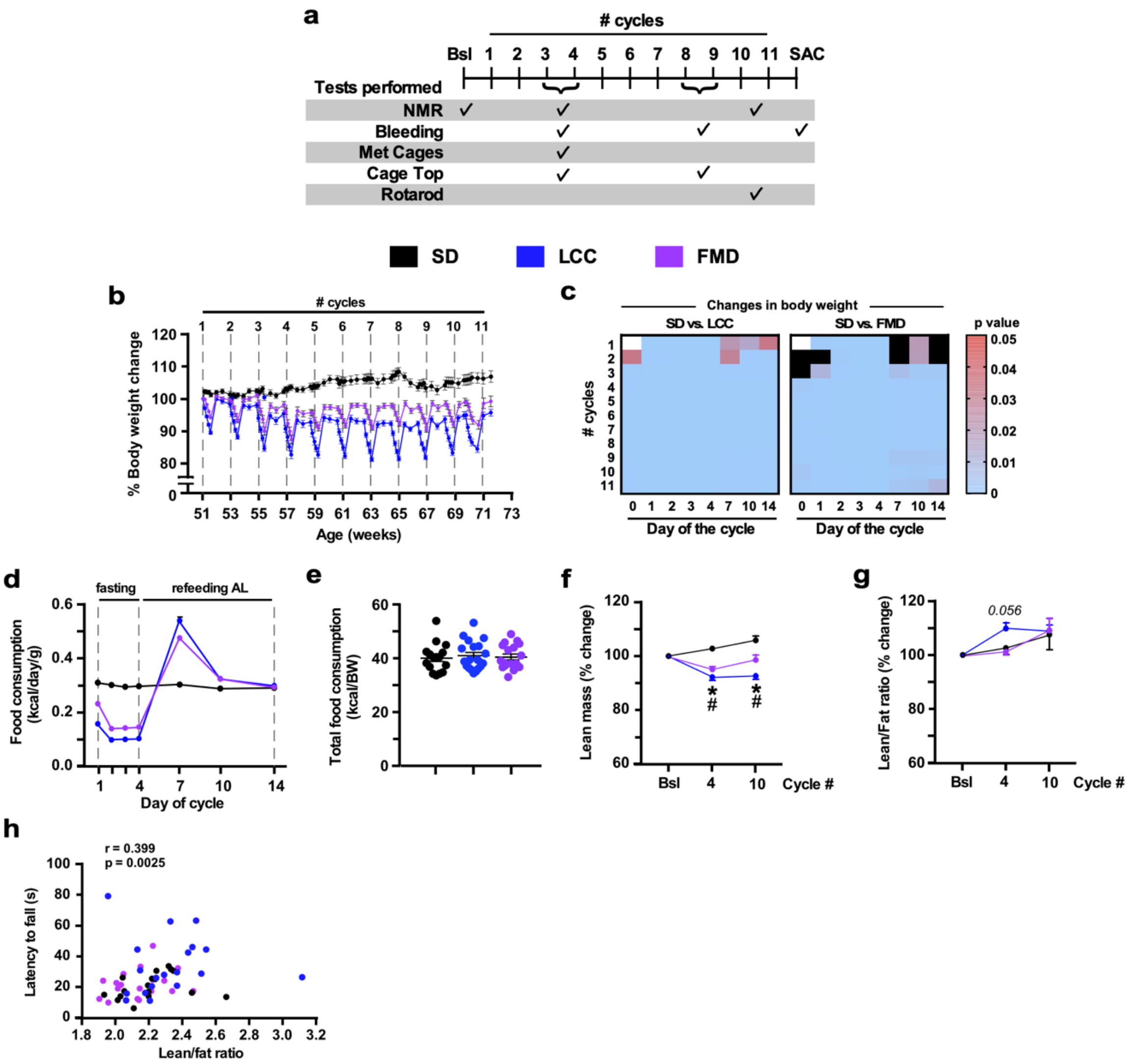

Supplementary Fig. 1, related to Fig. 1. Impact of 4:10 feeding cycles on physiological measurements in mice. **a** Timeline for procedures and functional tests. Bsl, baseline; SAC, sacrifice. **b-h** *Ad libitum* standard diet (SD, black symbols), LCC (blue symbols), and FMD (purple symbols). **b** High-resolution body weight (BW) trajectories over the course of the study (SD, n = 16-18; LCC, n = 19; FMD, n = 17-18); **c** Significant difference in body weight between AL and 4:10 groups of mice (LCC, left panel; FMD, right panel) at each day of the cycle for a total of 11 cycles, ranging from orange color, $p < 0.05$, to blue color, $p < 0.001$; not significant (black); **d** Average daily food consumption (kCal) during each cycle (n = 11 cycles) normalized to body weight; **e** Cumulative food consumption throughout the study after normalization to body weight (SD, n = 16; LCC, n = 19; FMD, n = 18). **f**, **g** Percent changes in **f** lean

mass content and **g** lean-to-fat ratio from baseline at cycles 4 and 10. Comparison by two-tailed t test; *$p < 0.05$ vs. SD. Measurements were carried out during the re-fed period of the indicated cycle (SD, n = 15/15/13; LCC, n = 17/17/15; FMD, n = 16). **h** Spearman correlation between latency to fall during cage top test and lean-to-fat ratio at cycle 4. n= 13-17 per group. All data are expressed as mean +/- SEM.

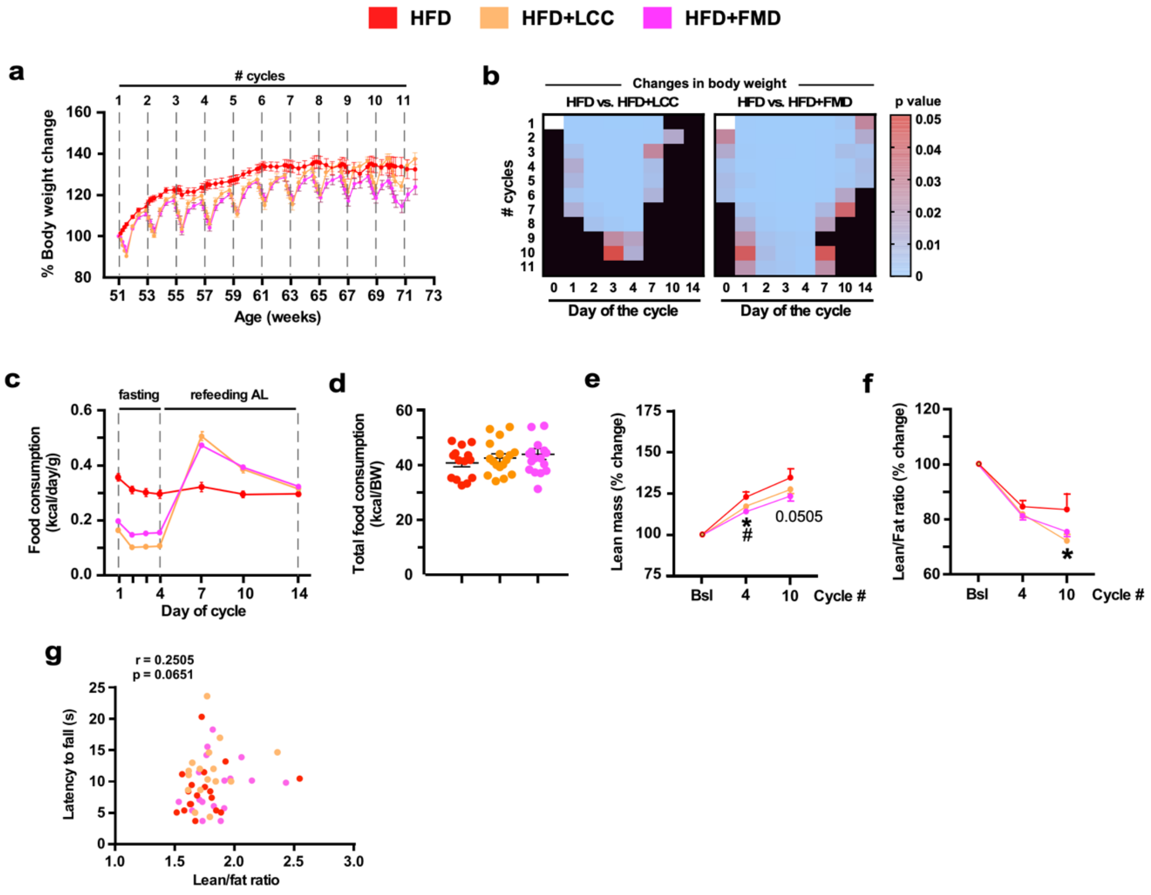

Supplementary Fig. 2, related to Fig. 2. Impact of 4:10 feeding cycles on physiological measurements in mice maintained on HFD. a-g *Ad libitum* HFD (red symbols), HFD+LCC (orange symbols), and HFD+FMD (pink symbols). **a** High-resolution BW trajectories over the course of the study (HDF, n = 16-19; HFD+LCC and HFD+FMD, n = 17-18 per group); **b** Significant difference in body weight between AL and 4:10 groups (HFD+LCC, left panel; HFD+FMD, right panel) at each day over the course of the study, ranging from orange color, p < 0.05, to blue color, p < 0.001; not significant (black). **c** Average daily food consumption (kCal) during each cycle (n = 11 cycles) normalized to body weight. **d** Cumulative food consumption throughout the study after normalization to body weight (HFD, n = 14; HFD+LCC, n = 17; HFD+FMD, n = 18). **e** Percent changes in lean mass content and **f** lean-to-fat ratio from baseline at cycles 4 and 10. Comparison by two-tailed t-test; *p< 0.05 vs. HFD (HFD, n= 17/16/13; HFD+LCC, n = 15/15/13; HFD+FMD, n = 14/14/11). Measurements were carried out during the re-fed period of the indicated cycle. **g** Spearman correlation between latency to fall during cage top test and lean-to-fat ratio at cycle 4. All data are expressed as mean ± SEM.

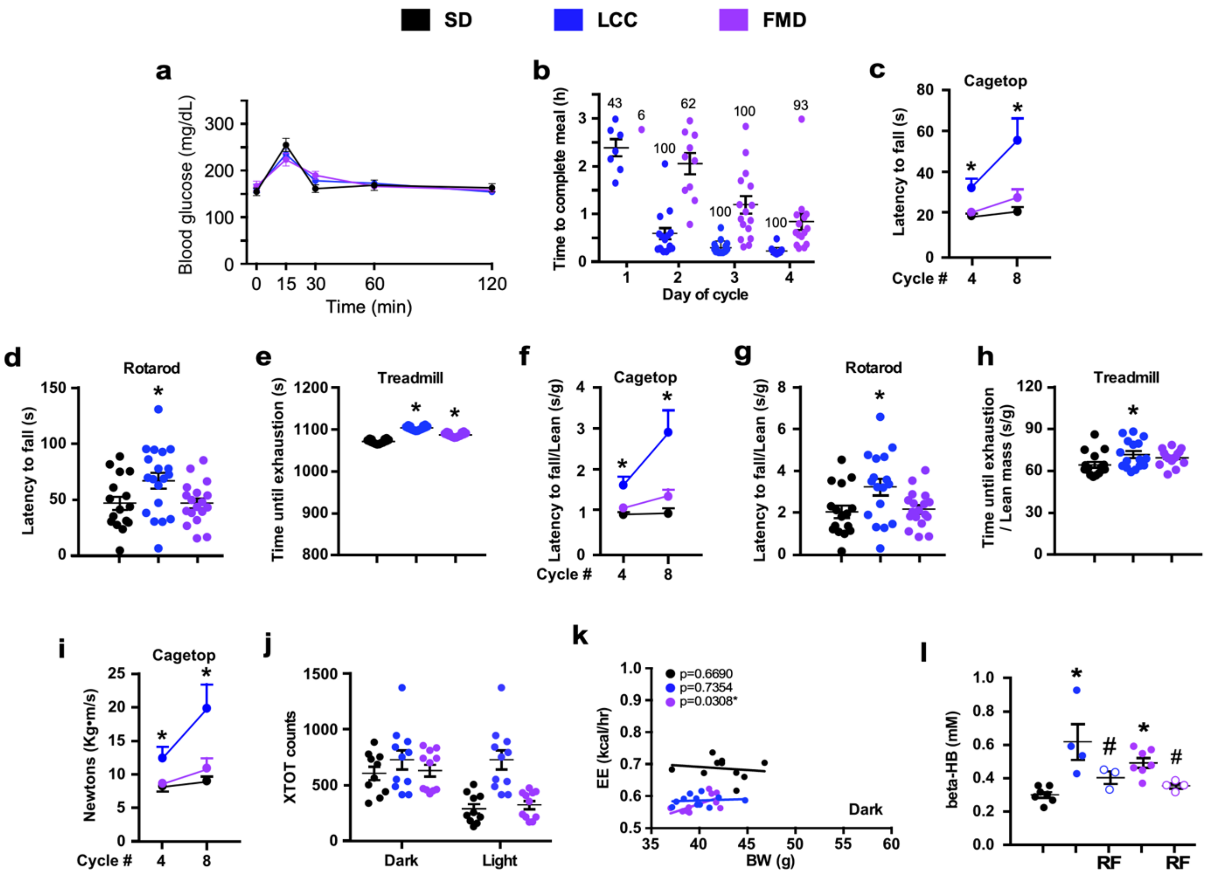

Supplementary Fig. 3, related to Fig. 3. Impact of 4:10 feeding cycles on metabolic and biochemical readouts in mice maintained on SD. **a** Blood glucose levels were measured over a period of 2 h during an OGTT, n=8 per group. OGTT was performed during the refeeding period. **b** Time required to eat daily portion of food on days 1-4 of the second cycle. Observations were made every two min for 3 h. Data was collected from an independent cohort of 20-week-old C57BL6/J male mice on LCC and FMD diet (LCC, n = 7 for day 1 and 16 for days 2-4; FMD, n = 1, 10, 16 and 15 for days 1-4, respectively). **c-e** All physical performance values were expressed as latency to fall (seconds) without body weight normalization. **c** Inverted cage top tests were conducted on days 10-13 of cycles 4 and 8 (SD, n = 18/17; LCC, n = 19/19; FMD, n = 18/18); **d** Rotarod tests were conducted on day 11 of cycle 8. (SD, n = 16; LCC, n = 19; FMD, n = 18); **e** Treadmill endurance test during the re-feeding period of cycle. Data were collected from an independent cohort of mice (n = 16) at the age of 26 weeks. **f-h** All physical performance values were expressed as latency to fall (seconds) and normalized by lean body mass in grams or **i** expressed as kg-m/sec - also known as newtons. **f** Inverted cage top tests (SD, n = 18/16; LCC, n = 19/17; FMD, n = 18/18). **g** Rotarod tests (SD, n = 16; LCC, n = 17; FMD, n = 18). **h** Treadmill endurance tests (n = 16). **i**, Inverted cage top tests expressed kg*m/sec – also known as newtons (SD, n =

18/17; LCC, n = 19/19; FMD, n = 18/18). **j, k** Mice were placed into metabolic cages during cycles 3 and 4 to measure **j** average activity counts in the X-axis and **k** Energy Expenditure (EE), which was then plotted in function of body weight (BW in grams) and analyzed by ANCOVA (SD, n = 10; LCC and FMD, n = 11). *$p < 0.05$. **l** β-hydroxybutyrate levels measured from serum collected at the time of sacrifice (sac). (SD, n = 7; LCC, n=4; LCC-RF, n=3; FMD, n=7; FMD-RF, n=4). All data are expressed as mean ± SEM. Comparison by one-way ANOVA unless otherwise noted; *, $p < 0.05$ vs. SD; #, $p < 0.05$ vs. LCC or FMD, respectively.

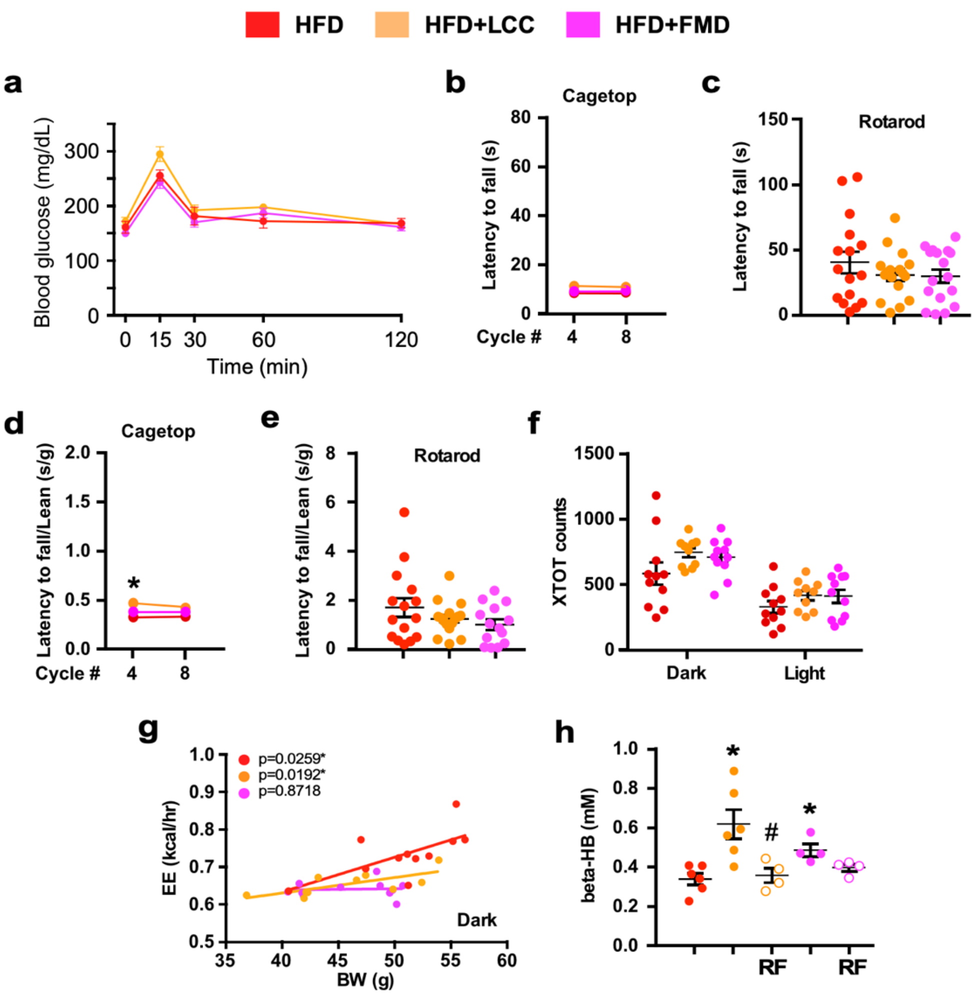

Supplementary Fig. 4, related to Fig. 4. Impact of 4:10 feeding cycles on metabolic and biochemical readouts in mice maintained on HFD. **a** Blood glucose levels were measured over a period of 2 h during an OGTT, n=8 per group. OGTT was performed during the refeeding period. **b, c** All physical performance values were expressed as latency to fall (seconds) without body weight normalization. **b** Inverted cage top tests were conducted on days 10-13 of cycles 4 and 8 (HFD, HFD+LCC and HFD+FMD, n = 18/17 per group); **c** Rotarod tests were conducted on day 11 of cycle 8. (HFD, n = 16; HFD+LCC, n = 17; HFD+FMD, n = 17). **d, e** All physical performance values were expressed as latency to fall (seconds) and normalized by lean body mass in grams. **d** Inverted cage top tests (HFD, n = 18/15; HFD+LCC, n = 18/16; HFD+FMD, n = 18/14). **e** Rotarod tests (HFD, HFD+LCC and HFD+FMD, n = 16 per group). **f, g** Mice fed HFD were placed into metabolic cages during cycles 3 and 4 to measure **f** average activity counts in the X-axis and **g** Energy Expenditure (EE), which was then plotted in function of body weight (BW in grams) and analyzed by ANCOVA (HFD, n = 11; HFD+LCC, n = 10;

HFD+FMD, n = 11). *p <0.05. **h** β-hydroxybutyrate levels measured from serum collected at the time of sacrifice (sac) (HFD, n = 6; HFD+LCC, n = 6; HFD+LCC-RF, n=4; HFD+FMD and HFD+FMD-RF, n = 4). All data are expressed as mean ± SEM. Comparison by one-way ANOVA unless otherwise noted; *, p <0.05 vs. SD; #, p < 0.05 vs. LCC or FMD, respectively.

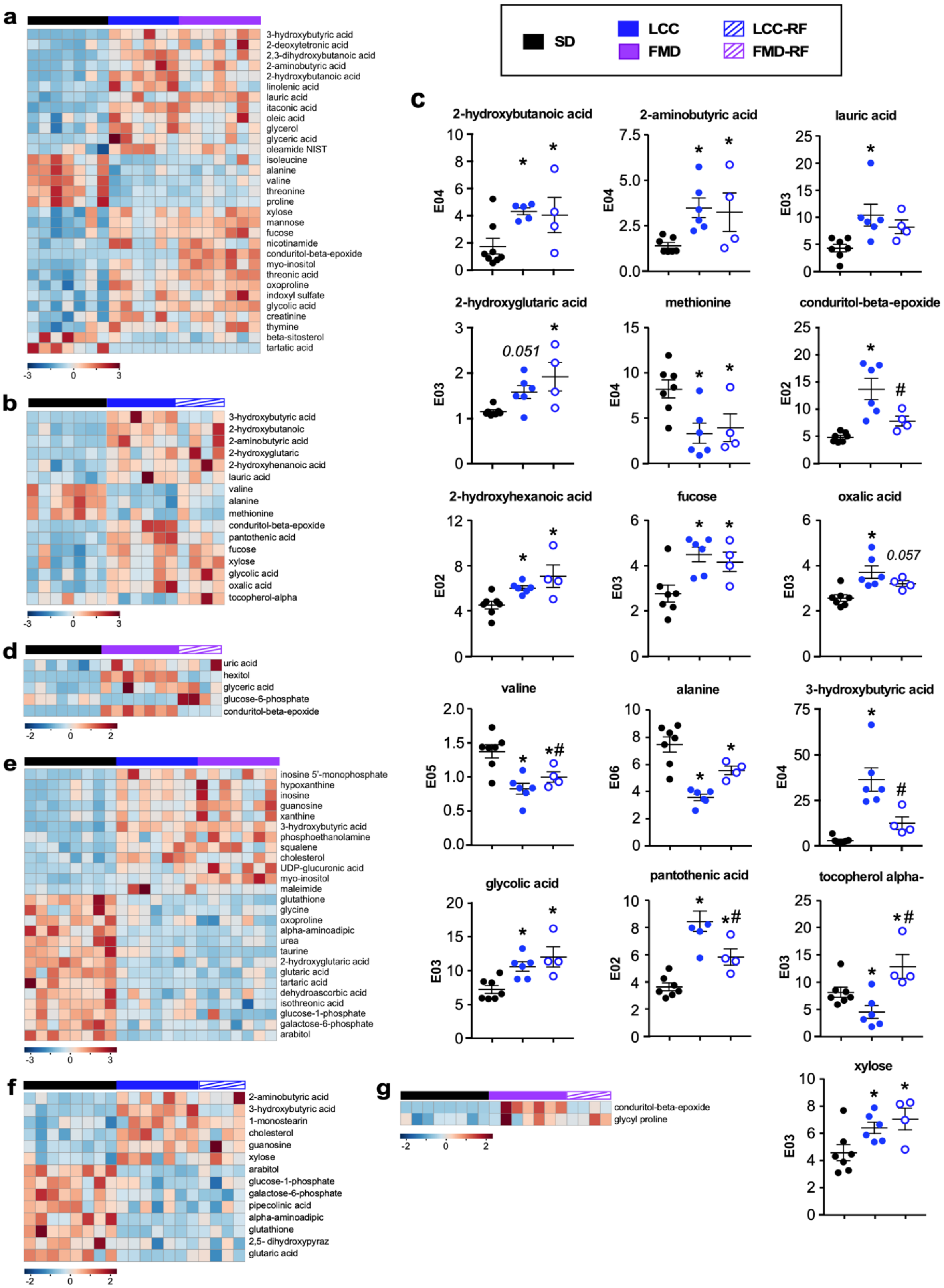
a
3-hydroxybutyric acid
2-deoxytetronic acid
2,3-dihydroxybutanoic acid
2-aminobutyric acid
2-hydroxybutanoic acid
linolenic acid
lauric acid
itaconic acid
oleic acid
glycerol
glyceric acid
oleamide NIST
isoleucine
alanine
valine
threonine
proline
xylose
mannose
fucose
nicotinamide
conduritol-beta-epoxide
myo-inositol
threonic acid
oxoproline
indoxyl sulfate
glycolic acid
creatinine
thymine
beta-sitosterol
tartatic acid
b
3-hydroxybutyric acid
2-hydroxybutanoic
2-aminobutyric acid
2-hydroxyglutaric
2-hydroxyhenanoic acid
lauric acid
valine
alanine
methionine
conduritol-beta-epoxide
pantothenic acid
fucose
xylose
glycolic acid
oxalic acid
tocopherol-alpha
d
uric acid
hexitol
glyceric acid
glucose-6-phosphate
conduritol-beta-epoxide
e
inosine 5'-monophosphate
hypoxanthine
inosine
guanosine
xanthine
3-hydroxybutyric acid
phosphoethanolamine
squalene
cholesterol
UDP-glucuronic acid
myo-inositol
maleimide
glutathione
glycine
oxoproline
alpha-aminoadipic
urea
taurine
2-hydroxyglutaric acid
glutaric acid
tartaric acid
dehydroascorbic acid
isothreonic acid
glucose-1-phosphate
galactose-6-phosphate
arabitol
f
2-aminobutyric acid
3-hydroxybutyric acid
1-monostearin
cholesterol
guanosine
xylose
arabitol
glucose-1-phosphate
galactose-6-phosphate
pipecolinic acid
alpha-aminoadipic
glutathione
2,5- dihydroxypyraz
glutaric acid
g
conduritol-beta-epoxide
glycyl proline
SD
LCC
FMD
LCC-RF
FMD-RF
c
2-hydroxybutanoic acid
2-aminobutyric acid
lauric acid
2-hydroxyglutaric acid
methionine
conduritol-beta-epoxide
2-hydroxyhexanoic acid
fucose
oxalic acid
valine
alanine
3-hydroxybutyric acid
glycolic acid
pantothenic acid
tocopherol alpha-
xylose
E02
E03
E04
E05
E06
0.051
0.057

Supplementary Fig. 5, related to Fig. 5 and 6. Heatmaps of metabolite profiles in serum and liver from mice on SD. **a-d** Untargeted metabolite profiling performed in serum of mice on SD. Metabolites that account for the separation between the metabolite profiles obtained from the Volcano plots were used to generate heatmaps. These heatmaps depict relative metabolite levels of individual animals. SD-fed mice on AL (black bar, n=7), LCC and FMD mice after 3 days of severe calorie restriction (blue/purple bar, n=6/7), LCC-RF and FMD-RF animals after 6 days of refeeding (hatched blue/purple bar, n=4/4). The color coding denotes the range of relative accumulation (red) to depletion (blue) for each metabolite. **a** Related to Fig. 3**d**, 'central core of fasting' in serum; **b, c** Related to Fig. 3**h**, 'selective metabolic memory' in LCC mice. Relative quantitation of selected metabolites in panel **c** were compared by one-way ANOVA. Results are expressed as mean ± SEM. *, $p < 0.05$ vs. SD; #, $p < 0.05$ vs. LCC; **d** Related to Fig. 3**j**, FMD mice. **e-g** Untargeted metabolite profiling performed in livers of mice on SD. SD-fed mice on AL (black bar, n=8), LCC and FMD mice after 3 days of severe calorie restriction (blue/purple bar, n=7/7), LCC-RF and FMD-RF animals after 6 days of refeeding (hatched blue/purple bar, n=4/4). **e** Related Fig. 3**n**, 'central core of fasting' in liver; **f** Related to Fig. 3**r**, 'selective metabolic memory' in LCC mice; **g** Related to Fig. 3**t**, FMD mice.

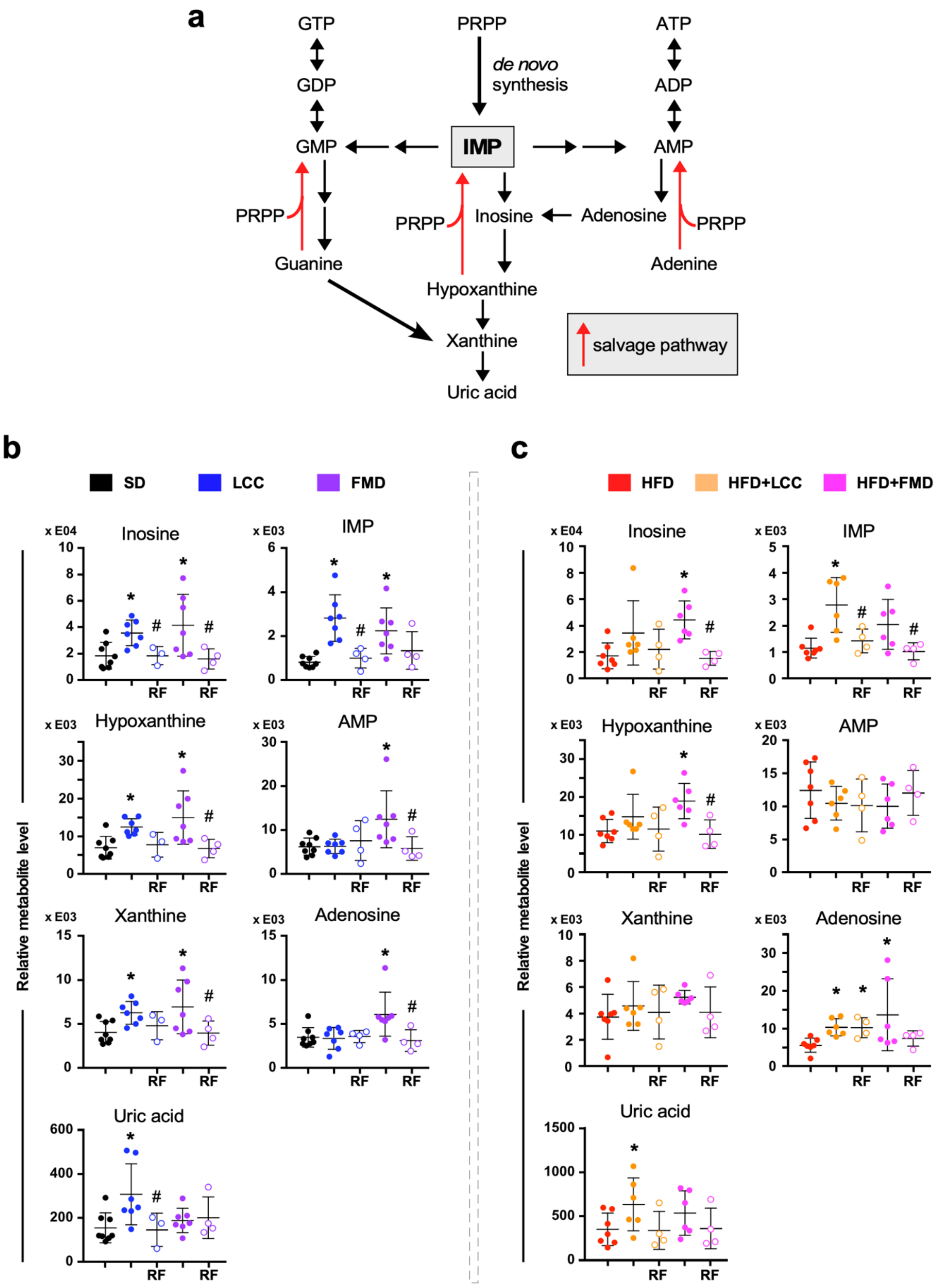
a
GTP
GDP
GMP
PRPP
de novo synthesis
IMP
ATP
ADP
AMP
PRPP
Guanine
PRPP
Inosine
Adenosine
PRPP
Adenine
Hypoxanthine
Xanthine
Uric acid
salvage pathway
b
SD
LCC
FMD
Relative metabolite level
Inosine
IMP
Hypoxanthine
AMP
Xanthine
Adenosine
Uric acid
RF
c
HFD
HFD+LCC
HFD+FMD
Relative metabolite level
Inosine
IMP
Hypoxanthine
AMP
Xanthine
Adenosine
Uric acid
RF

Supplementary Fig. 6, related to Fig. 6 and 8. Relative quantitation of metabolites related to purine metabolism in liver from mice on SD and HFD. **a** Schematic representation of de novo and salvage pathways for purine synthesis. **b**, **c** One-way ANOVA analysis of relative levels of selected metabolites in liver from mice on **b** SD and **c** HFD diet under three feeding regimens. AL feeding (SD and HFD), 3 days of severe calorie restriction (LCC, FMD, HFD+LCC and HFD+FMD), and 6 days of refeeding (LCC-RF, FMD-RF, HFD+LCC-RF and HFD+FMD-RF). SD, n = 8; LCC, n=7; LCC-RF, n=3-4; FMD, n = 7; FMD-RF, n=4; HFD, n = 7; HFD+LCC, n = 6; HFD+LCC-RF, n=4; HFD+FMD, n = 6; HFD+FMD-RF, n=4). Results are expressed as mean ± SD. *, $p < 0.05$ vs. SD or HFD; #, $p < 0.05$ vs. LCC, FMD, HFD+LCC or HFD+FMD.

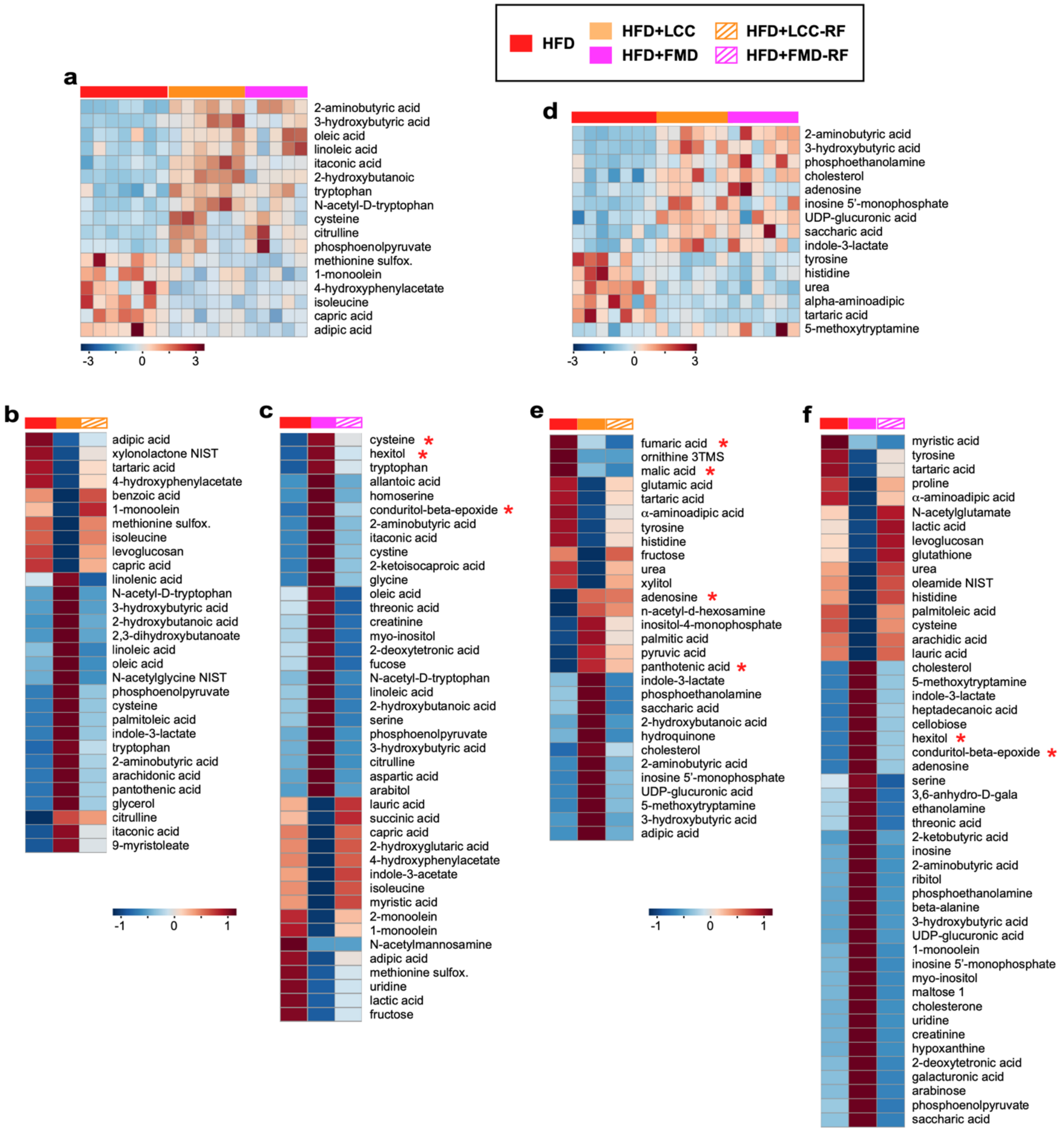

Supplementary Fig. 7, related to Fig. 7 and 8. Heatmaps of metabolite profiles in serum and liver from mice on HFD. **a-c** Untargeted metabolite profiling was performed in serum and liver of mice on HFD. Metabolites that account for the separation between the metabolite profiles obtained from the Volcano plots were used to generate heatmaps. Heatmaps depict relative metabolite levels of **a** individual animals or **b**, **c** averaged signal. HFD-fed mice on AL (red bar, n=7), LCC and FMD mice on HFD after 3 days of severe calorie restriction (tangerine/magenta bar, n=6/5), LCC-RF and FMD-RF on HFD after 6 days of refeeding (hatched tangerine/magenta bar, n=4/4). The color coding denotes the range of relative

accumulation (red) to depletion (blue) for each metabolite. **a** Related to Fig 4**d**, ‘central core of fasting’ in serum; **b, c** Related to Figs. 4**a** and 4**b**, metabolites impacted by 3 days of severe low-calorie intake in **b** HFD+LCC or **c** HFD+FMD mice. Red asterisks indicate metabolites also present after refeeding. **d-f** Untargeted metabolite profiling performed in livers of mice on HFD. HFD-fed mice on AL (red bar, n=7), LCC and FMD mice on HFD after 3 days of severe calorie restriction (tangerine/magenta bar, n=6/6), LCC-RF and FMD-RF on HFD after 6 days of refeeding (hatched tangerine/magenta bar, n=4/4). **d** Related to Fig. 4**l**, ‘central core of fasting’ in liver; **e, f** Related to Figs. 4**i** and 4**j**, hepatic metabolites impacted by 3 days of severe low-calorie intake in **e** HFD+LCC mice or **f** HFD+FMD mice. Red asterisks indicate metabolites also present after refeeding.

Note regarding Supplementary Tables 1-3: 194 metabolites were analyzed using MetaboAnalyst versions 3.0 and 4.0 (31, 56). Significantly changed metabolites were determined from the ratio LCC/AL or refeeding/AL both in SD or FMD using as threshold (fold-change up ≥ 1.2 or down ≤ 0.83, raw p value < 0.05). The same procedure was utilized in HFD diet. Supplementary Table 4: Pathway analysis was carried out using MetaboAnalyst 4.0. Significantly changed pathways were determined using a threshold (log $p > 2.5$

Supplementary Table 1. List of serum and liver metabolites significantly impacted by LCC and FMD in mice fed SD and HFD.

Biological Sample	*Pairwise Comparison*	*Metabolite*	*FC*	*log2(FC)*	*raw.pval*	*-LOG10(p)*
Serum	LCC vs SD	3-hydroxybutyric acid	14.04	3.81	1.59E-04	3.80
Serum		linolenic acid	4.57	2.19	1.03E-03	2.99
Serum		hypoxanthine	4.18	2.06	9.67E-03	2.01
Serum		2,3-dihydroxybutanoic acid NIST	3.55	1.83	7.74E-06	5.11
Serum		xanthine	3.46	1.79	2.14E-02	1.67
Serum		2-hydroxybutanoic acid	3.26	1.70	1.58E-05	4.80
Serum		cholesterone	2.95	1.56	2.28E-02	1.64
Serum		conduritol-beta-expoxide	2.79	1.48	4.59E-04	3.34
Serum		lauric acid	2.65	1.41	1.18E-02	1.93
Serum		itaconic acid	2.60	1.38	3.20E-06	5.50
Serum		2-aminobutyric acid	2.40	1.26	2.44E-03	2.61
Serum		indole-3-lactate	2.34	1.23	3.65E-06	5.44
Serum		pantothenic acid	2.29	1.20	5.54E-05	4.26
Serum		palmitoleic acid	2.21	1.14	7.98E-03	2.10
Serum		myo-inositol	2.07	1.05	2.65E-03	2.58
Serum		ribonic acid	1.98	0.98	1.02E-02	1.99
Serum		mannose	1.81	0.85	1.99E-02	1.70
Serum		9-myristoleate	1.80	0.85	7.14E-03	2.15
Serum		glyceric acid	1.78	0.83	1.30E-02	1.89
Serum		oleic acid	1.77	0.83	1.08E-02	1.97
Serum		indoxyl sulfate	1.76	0.81	3.76E-03	2.42
Serum		creatinine	1.75	0.81	7.96E-03	2.10
Serum		fucose	1.63	0.70	5.86E-03	2.23
Serum		urocanic acid	1.56	0.64	2.87E-02	1.54
Serum		2-deoxytetronic acid	1.55	0.63	1.40E-02	1.85
Serum		nicotinamide	1.52	0.60	3.49E-02	1.46
Serum		glycerol	1.52	0.60	6.00E-03	2.22
Serum		galactonic acid	1.51	0.60	2.27E-02	1.64
Serum		threonic acid	1.49	0.58	2.84E-03	2.55
Serum		glycolic acid	1.48	0.56	3.15E-03	2.50
Serum		oleamide NIST	1.47	0.56	1.60E-02	1.80
Serum		oxalic acid	1.45	0.54	2.81E-03	2.55
Serum		thymine	1.45	0.53	2.73E-02	1.56
Serum		xylose	1.40	0.48	3.30E-02	1.48
Serum		oxoproline	1.38	0.47	2.79E-03	2.56
Serum		2-hydroxyglutaric acid	1.37	0.46	9.50E-03	2.02
Serum		2-hydroxyhexanoic acid	1.34	0.43	3.91E-03	2.41
Serum		N-acetyl-D-tryptophan	1.28	0.35	4.20E-02	1.38
Serum		glutamine	0.82	-0.29	4.01E-02	1.40
Serum		alpha-aminoadipic acid	0.74	-0.43	2.14E-02	1.67
Serum		leucine	0.70	-0.52	1.25E-02	1.90
Serum		tyrosine	0.70	-0.52	3.79E-02	1.42
Serum		isoleucine	0.61	-0.71	4.50E-04	3.35
Serum		alanine	0.60	-0.74	1.30E-03	2.89
Serum		4-hydroxyphenylacetic acid	0.58	-0.78	4.66E-02	1.33
Serum		tocopherol alpha-	0.56	-0.84	3.23E-02	1.49
Serum		threonine	0.53	-0.92	8.48E-04	3.07
Serum		beta-sitosterol	0.51	-0.97	2.03E-02	1.69
Serum		valine	0.48	-1.07	8.28E-05	4.08
Serum		methionine	0.41	-1.28	7.30E-03	2.14
Serum		proline	0.31	-1.67	3.97E-03	2.40
Serum		tartaric acid	0.04	-4.56	5.58E-03	2.25
Serum		conduritol-beta-expoxide	27.41	4.78	4.02E-09	8.40
Serum		hexitol	14.65	3.87	1.28E-09	8.89
Serum		3-hydroxybutyric acid	14.34	3.84	1.08E-06	5.97
Serum		lauric acid	4.18	2.06	1.26E-07	6.90
Serum		myo-inositol	3.44	1.78	3.46E-06	5.46
Serum		2,3-dihydroxybutanoic acid NIST	2.69	1.43	3.36E-04	3.47
Serum		allantoic acid	2.55	1.35	6.13E-03	2.21
Serum		linolenic acid	2.33	1.22	4.45E-02	1.35
Serum		mannose	2.32	1.21	3.38E-04	3.47
Serum		itaconic acid	2.16	1.11	6.58E-04	3.18
Serum		2-aminobutyric acid	2.15	1.11	5.92E-04	3.23
Serum		gluconic acid	2.01	1.00	4.32E-04	3.37
Serum		2-hydroxybutanoic acid	1.97	0.98	1.00E-02	2.00
Serum		fucose	1.94	0.95	4.17E-04	3.38
Serum		indoxyl sulfate	1.90	0.93	5.78E-03	2.24
Serum		isohexonic acid	1.85	0.89	3.01E-04	3.52
Serum		saccharic acid	1.85	0.89	1.35E-03	2.87
Serum		threonic acid	1.82	0.86	1.38E-05	4.86
Serum		2-deoxytetronic acid	1.80	0.85	1.97E-02	1.71

Serum	FMD vs. SD	citric acid	1.77	0.82	9.27E-04	3.03
Serum		glutathione	1.66	0.73	1.19E-02	1.93
Serum		glycolic acid	1.62	0.69	4.63E-05	4.33
Serum		sorbitol	1.61	0.68	3.89E-02	1.41
Serum		cystine	1.61	0.68	1.87E-02	1.73
Serum		isocitric acid	1.61	0.68	6.71E-03	2.17
Serum		phosphoethanolamine	1.60	0.68	4.59E-02	1.34
Serum		xylitol	1.60	0.68	4.78E-02	1.32
Serum		oleic acid	1.60	0.67	4.13E-02	1.38
Serum		nicotinamide	1.56	0.64	2.61E-03	2.58
Serum		pseudo uridine	1.54	0.62	1.76E-02	1.75
Serum		creatinine	1.54	0.62	1.27E-02	1.89
Serum		xylose	1.48	0.57	9.92E-03	2.00
Serum		2-ketobutyric acid	1.48	0.56	1.28E-03	2.89
Serum		thymine	1.46	0.54	3.23E-02	1.49
Serum		N-acetylglycine NIST	1.40	0.48	2.00E-02	1.70
Serum		oxoproline	1.39	0.47	2.51E-04	3.60
Serum		N-acetylglutamate	1.37	0.45	1.61E-04	3.79
Serum		glyceric acid	1.36	0.44	1.59E-02	1.80
Serum		trans-4-hydroxy-L-proline	1.35	0.43	8.19E-03	2.09
Serum		glycerol	1.33	0.41	1.43E-02	1.84
Serum		uric acid	1.32	0.40	4.97E-03	2.30
Serum		oleamide NIST	1.31	0.39	4.09E-02	1.39
Serum		ethanol phosphate NIST	1.31	0.39	4.89E-02	1.31
Serum		ethanolamine	1.27	0.34	3.56E-02	1.45
Serum		isoleucine	0.76	-0.40	2.87E-02	1.54
Serum		alanine	0.63	-0.66	8.58E-04	3.07
Serum		valine	0.61	-0.71	6.13E-04	3.21
Serum		threonine	0.61	-0.71	1.49E-03	2.83
Serum		beta-sitosterol	0.53	-0.92	1.99E-02	1.70
Serum		dihydrocholesterol	0.40	-1.33	1.86E-02	1.73
Serum		proline	0.39	-1.36	4.35E-03	2.36
Serum		glucose-6-phosphate	0.31	-1.71	1.32E-03	2.88
Serum		p-tolyl glucuronide	0.22	-2.21	5.03E-03	2.30
Serum		tartaric acid	0.04	-4.62	2.82E-03	2.55
Serum	LCCRF vs. SD	3-hydroxybutyric acid	4.72	2.24	5.97E-03	2.22
Serum		2-hydroxybutanoic acid	2.97	1.57	2.56E-02	1.59
Serum		2-aminobutyric acid	2.24	1.16	4.68E-02	1.33
Serum		lauric acid	2.07	1.05	1.50E-02	1.83
Serum		sorbitol	1.74	0.80	4.96E-02	1.30
Serum		glycolic acid	1.68	0.75	5.76E-03	2.24
Serum		2-hydroxyglutaric acid	1.66	0.73	9.35E-03	2.03
Serum		2-ketobutyric acid	1.63	0.70	5.11E-03	2.29
Serum		conduritol-beta-expoxide	1.60	0.68	4.48E-03	2.35
Serum		pantothenic acid	1.59	0.67	4.60E-03	2.34
Serum		2-hydroxyhexanoic acid	1.57	0.66	1.54E-02	1.81
Serum		tocopherol alpha-	1.57	0.65	4.36E-02	1.36
Serum		xylose	1.54	0.62	3.42E-02	1.47
Serum		fucose	1.51	0.60	4.31E-02	1.37
Serum		cholesterol	1.49	0.57	1.41E-02	1.85
Serum		uric acid	1.37	0.46	3.22E-02	1.49
Serum		methionine sulfoxide	1.33	0.41	4.30E-02	1.37
Serum		saccharic acid	1.30	0.37	2.61E-02	1.58
Serum		oxalic acid	1.25	0.32	1.65E-02	1.78
Serum		valine	0.74	-0.43	3.89E-02	1.41
Serum		alanine	0.72	-0.46	2.68E-02	1.57
Serum		methionine	0.49	-1.04	3.69E-02	1.43
Serum	FMDRF vs. SD	conduritol-beta-expoxide	7.27	2.86	2.58E-06	5.59
Serum		6-deoxyglucose	3.47	1.79	2.60E-02	1.58
Serum		hexitol	3.13	1.65	1.86E-02	1.73
Serum		glucose-6-phosphate	2.25	1.17	1.91E-02	1.72
Serum		9-myristoleate	1.78	0.83	1.55E-02	1.81
Serum		uric acid	1.34	0.42	3.96E-02	1.40
Serum		glyceric acid	1.31	0.39	3.19E-02	1.50
Liver		3-hydroxybutyric acid	4.86	2.28	1.82E-06	5.74
Liver		inosine 5'-monophosphate	3.42	1.78	1.68E-04	3.77
Liver		oleic acid	3.31	1.73	3.52E-02	1.45
Liver		squalene	2.82	1.50	4.20E-04	3.38
Liver		2-aminobutyric acid	2.51	1.33	3.06E-03	2.51
Liver		maleimide	2.11	1.07	1.86E-02	1.73
Liver		uric acid	1.99	0.99	1.62E-02	1.79
Liver		inosine	1.93	0.95	5.07E-03	2.30
Liver		hypoxanthine	1.80	0.85	1.46E-03	2.84
Liver		1-monostearin	1.66	0.73	7.58E-03	2.12
Liver		linolenic acid	1.64	0.72	2.57E-02	1.59

Liver		guanosine	1.61	0.68	3.60E-05	4.44
Liver		UDP-glucuronic acid	1.57	0.65	5.64E-03	2.25
Liver		xanthine	1.56	0.64	4.93E-03	2.31
Liver		cholesterol	1.51	0.59	4.80E-04	3.32
Liver		1-monopalmitin	1.50	0.58	4.55E-02	1.34
Liver		cytidine-5-monophosphate	1.46	0.54	1.49E-03	2.83
Liver		myo-inositol	1.43	0.52	2.65E-03	2.58
Liver		phosphoethanolamine	1.42	0.50	3.38E-02	1.47
Liver		xanthosine	1.36	0.44	4.23E-02	1.37
Liver		xylose	1.24	0.31	3.92E-02	1.41
Liver		oxoproline	0.84	-0.25	1.44E-02	1.84
Liver		isoleucine	0.80	-0.31	2.86E-02	1.54
Liver	LCC vs SD	dehydroascorbic acid	0.78	-0.35	1.63E-02	1.79
Liver		glycine	0.76	-0.39	2.80E-02	1.55
Liver		aminomalonate	0.75	-0.42	4.93E-02	1.31
Liver		isothreonic acid	0.75	-0.42	8.01E-03	2.10
Liver		2,5-dihydroxypyrazine NIST	0.74	-0.43	1.77E-02	1.75
Liver		pipecolinic acid	0.68	-0.56	1.05E-02	1.98
Liver		fumaric acid	0.67	-0.57	2.87E-02	1.54
Liver		glucose-1-phosphate	0.67	-0.57	5.42E-03	2.27
Liver		UDP-N-acetylglucosamine	0.66	-0.61	1.07E-02	1.97
Liver		citrulline	0.64	-0.65	3.34E-02	1.48
Liver		glutaric acid	0.61	-0.71	6.27E-04	3.20
Liver		urea	0.60	-0.75	5.62E-04	3.25
Liver		N-acetylglutamate	0.55	-0.85	1.72E-02	1.76
Liver		malic acid	0.55	-0.86	1.18E-02	1.93
Liver		galactose-6-phosphate	0.54	-0.89	2.59E-03	2.59
Liver		taurine	0.54	-0.90	1.24E-02	1.91
Liver		9-myristoleate	0.52	-0.95	4.05E-02	1.39
Liver		2-hydroxyglutaric acid	0.48	-1.05	4.29E-07	6.37
Liver		arabitol	0.44	-1.20	1.52E-05	4.82
Liver		uridine	0.41	-1.30	1.30E-02	1.89
Liver		glutathione	0.35	-1.51	3.24E-04	3.49
Liver		tartaric acid	0.32	-1.63	1.63E-03	2.79
Liver		alpha-aminoadipic acid	0.31	-1.70	1.02E-03	2.99
Liver		conduritol-beta-expoxide	12.54	3.65	7.11E-05	4.15
Liver		3-hydroxybutyric acid	4.78	2.26	2.81E-07	6.55
Liver		squalene	3.03	1.60	2.71E-03	2.57
Liver		inosine 5'-monophosphate	2.72	1.44	2.53E-03	2.60
Liver		myo-inositol	2.60	1.38	5.00E-06	5.30
Liver		ribose	2.52	1.33	4.19E-02	1.38
Liver		ethanolamine	2.37	1.25	8.04E-04	3.09
Liver		UDP-glucuronic acid	2.34	1.22	2.11E-03	2.67
Liver		inosine	2.25	1.17	2.48E-02	1.61
Liver		guanosine	2.17	1.12	1.40E-05	4.85
Liver		hypoxanthine	2.16	1.11	1.21E-02	1.92
Liver		arabinose	2.13	1.09	3.24E-02	1.49
Liver		isohexonic acid	2.05	1.03	8.38E-03	2.08
Liver		adenosine-5-monophosphate	2.01	1.01	2.18E-02	1.66
Liver		beta-alanine	1.94	0.96	2.07E-02	1.68
Liver		glycyl proline	1.78	0.83	2.78E-02	1.56
Liver		threonic acid	1.78	0.83	1.87E-02	1.73
Liver		adenosine	1.77	0.82	1.84E-02	1.74
Liver		xanthine	1.73	0.79	2.77E-02	1.56
Liver		allantoic acid	1.73	0.79	8.66E-03	2.06
Liver		inositol-4-monophosphate	1.73	0.79	3.20E-02	1.49
Liver		phosphoethanolamine	1.67	0.74	1.19E-02	1.92
Liver		phosphoenolpyruvate	1.66	0.73	1.61E-02	1.79
Liver		galacturonic acid	1.62	0.70	3.42E-03	2.47
Liver		N-acetylglycine NIST	1.62	0.70	4.01E-03	2.40
Liver		creatinine	1.59	0.67	7.15E-03	2.15
Liver		itaconic acid	1.58	0.66	1.57E-02	1.80
Liver	FMD vs. SD	glutamine	1.57	0.65	2.08E-02	1.68
Liver		gluconic acid	1.49	0.58	3.20E-02	1.49
Liver		serine	1.49	0.58	2.60E-04	3.59
Liver		5'-deoxy-5'-methylthioadenosine	1.47	0.56	3.94E-02	1.40
Liver		hexuronic acid	1.38	0.46	3.44E-02	1.46
Liver		phenylalanine	1.27	0.35	2.43E-02	1.61
Liver		cholesterol	1.26	0.33	1.16E-02	1.94
Liver		maleimide	1.24	0.31	1.39E-02	1.86
Liver		trans-4-hydroxy-L-proline	0.79	-0.34	7.04E-03	2.15
Liver		glycine	0.78	-0.36	2.87E-02	1.54
Liver		dehydroascorbic acid	0.77	-0.37	1.03E-02	1.99
Liver		oxoproline	0.75	-0.42	4.77E-03	2.32
Liver		dihydrocholesterol	0.74	-0.43	2.20E-02	1.66

Liver		arachidonic acid	0.70	-0.52	4.93E-02	1.31
Liver		urea	0.59	-0.76	6.71E-04	3.17
Liver		2-hydroxyglutaric acid	0.59	-0.77	4.31E-05	4.37
Liver		isothreonic acid	0.56	-0.83	3.92E-04	3.41
Liver		galactose-6-phosphate	0.55	-0.87	4.80E-03	2.32
Liver		glutaric acid	0.55	-0.87	6.22E-04	3.21
Liver		arabitol	0.52	-0.95	1.65E-04	3.78
Liver		ascorbic acid	0.49	-1.01	2.18E-02	1.66
Liver		glucose-1-phosphate	0.44	-1.20	4.58E-03	2.34
Liver		tartaric acid	0.31	-1.70	1.81E-03	2.74
Liver		alpha-aminoadipic acid	0.28	-1.86	8.79E-04	3.06
Liver		hydroxycarbamate NIST	0.26	-1.97	4.03E-02	1.39
Liver		glutathione	0.23	-2.13	9.61E-05	4.02
Liver		taurine	0.09	-3.40	1.79E-05	4.75
Liver		citric acid	3.87	1.95	3.43E-02	1.46
Liver		2-aminobutyric acid	2.84	1.51	1.63E-02	1.79
Liver		3-hydroxybutyric acid	2.25	1.17	3.39E-02	1.47
Liver		gluconic acid	2.10	1.07	2.41E-02	1.62
Liver		galactonic acid	1.99	0.99	3.58E-02	1.45
Liver		guanosine	1.66	0.73	4.09E-02	1.39
Liver		allantoic acid	1.53	0.62	4.53E-02	1.34
Liver		1-monostearin	1.53	0.61	1.60E-02	1.80
Liver		serine	1.29	0.36	1.32E-02	1.88
Liver		cholesterol	1.28	0.36	4.06E-02	1.39
Liver	LCCRF vs. SD	xylose	1.25	0.32	2.60E-02	1.58
Liver		behenic acid	0.77	-0.37	3.64E-02	1.44
Liver		2,5-dihydroxypyrazine NIST	0.72	-0.48	4.31E-02	1.37
Liver		pipecolinic acid	0.72	-0.48	3.52E-02	1.45
Liver		glucose-1-phosphate	0.66	-0.59	3.78E-02	1.42
Liver		galacturonic acid	0.64	-0.63	2.28E-02	1.64
Liver		glutaric acid	0.63	-0.68	2.10E-02	1.68
Liver		arabitol	0.57	-0.82	3.60E-03	2.44
Liver		glutathione	0.50	-1.01	1.98E-02	1.70
Liver		glycerol-alpha-phosphate	0.42	-1.25	3.95E-02	1.40
Liver		alpha-aminoadipic acid	0.41	-1.29	2.72E-02	1.57
Liver		galactose-6-phosphate	0.36	-1.48	1.96E-03	2.71
Liver		conduritol-beta-expoxide	4.22	2.08	1.79E-04	3.75
Liver		citric acid	2.95	1.56	1.22E-02	1.91
Liver		uracil	2.09	1.06	4.76E-02	1.32
Liver	FMDRF vs. SD	glycyl proline	1.79	0.84	1.58E-02	1.80
Liver		asparagine	0.80	-0.33	2.48E-02	1.61
Liver		N-acetylmannosamine	0.60	-0.73	3.03E-02	1.52
Liver		raffinose	0.43	-1.22	4.79E-02	1.32
Liver		p-tolyl glucuronide	0.41	-1.28	7.04E-03	2.15
Serum		3-hydroxybutyric acid	5.59	2.48	6.97E-04	3.16
Serum		2-aminobutyric acid	4.84	2.28	4.08E-06	5.39
Serum		2-hydroxybutanoic acid	3.87	1.95	4.16E-06	5.38
Serum		cysteine	3.36	1.75	2.21E-02	1.66
Serum		2,3-dihydroxybutanoic acid NIST	3.11	1.64	2.57E-04	3.59
Serum		itaconic acid	2.77	1.47	2.27E-05	4.64
Serum		N-acetyl-D-tryptophan	2.53	1.34	3.05E-04	3.52
Serum		N-acetylglycine NIST	2.23	1.16	3.27E-02	1.49
Serum		indole-3-lactate	2.22	1.15	1.93E-03	2.71
Serum		pantothenic acid	2.06	1.04	4.16E-02	1.38
Serum		citrulline	2.02	1.02	4.82E-02	1.32
Serum		tryptophan	1.73	0.79	3.06E-04	3.51
Serum		palmitoleic acid	1.63	0.71	2.95E-03	2.53
Serum		oleic acid	1.57	0.65	1.39E-02	1.86
Serum	HFD+LCC vs HFD	phosphoenolpyruvate	1.53	0.62	4.50E-02	1.35
Serum		linolenic acid	1.49	0.57	6.23E-03	2.21
Serum		linoleic acid	1.46	0.54	9.05E-03	2.04
Serum		arachidonic acid	1.44	0.52	1.40E-02	1.85
Serum		9-myristoleate	1.38	0.46	1.20E-02	1.92
Serum		glycerol	1.26	0.34	4.64E-02	1.33
Serum		benzoic acid	0.82	-0.28	2.30E-02	1.64
Serum		1-monoolein	0.71	-0.49	4.93E-02	1.31
Serum		isoleucine	0.64	-0.65	2.94E-03	2.53
Serum		levoglucosan	0.63	-0.67	1.92E-02	1.72
Serum		capric acid	0.57	-0.82	7.52E-03	2.12
Serum		adipic acid	0.55	-0.85	3.14E-02	1.50
Serum		methionine sulfoxide	0.51	-0.97	3.00E-02	1.52
Serum		4-hydroxyphenylacetic acid	0.49	-1.04	2.18E-02	1.66
Serum		xylonolactone NIST	0.36	-1.48	3.33E-02	1.48
Serum		tartaric acid	0.04	-4.48	3.30E-02	1.48
Serum		conduritol-beta-expoxide	10.20	3.35	1.08E-05	4.97

Serum	HFD+FMD vs HFD	hexitol	7.79	2.96	2.96E-03	2.53
Serum		2-aminobutyric acid	4.83	2.27	2.42E-04	3.62
Serum		aspartic acid	3.30	1.72	3.25E-02	1.49
Serum		3-hydroxybutyric acid	3.06	1.61	4.62E-03	2.34
Serum		allantoic acid	2.76	1.46	1.08E-02	1.97
Serum		2-ketoisocaproic acid	2.63	1.39	1.41E-02	1.85
Serum		arabitol	2.54	1.35	5.28E-04	3.28
Serum		cysteine	2.53	1.34	1.06E-02	1.97
Serum		citrulline	2.49	1.32	7.12E-03	2.15
Serum		2-hydroxybutanoic acid	2.06	1.04	1.74E-02	1.76
Serum		cystine	1.92	0.94	1.09E-04	3.96
Serum		creatinine	1.85	0.89	2.56E-02	1.59
Serum		phosphoenolpyruvate	1.77	0.82	4.27E-02	1.37
Serum		oleic acid	1.74	0.80	3.25E-02	1.49
Serum		myo-inositol	1.62	0.70	2.17E-02	1.66
Serum		linoleic acid	1.60	0.68	4.26E-02	1.37
Serum		tryptophan	1.57	0.65	7.58E-03	2.12
Serum		itaconic acid	1.56	0.65	6.50E-03	2.19
Serum		homoserine	1.52	0.61	7.01E-03	2.15
Serum		N-acetyl-D-tryptophan	1.52	0.60	3.32E-02	1.48
Serum		2-deoxytetronic acid	1.35	0.43	9.31E-03	2.03
Serum		fucose	1.33	0.42	3.98E-02	1.40
Serum		threonic acid	1.33	0.41	2.56E-02	1.59
Serum		glycine	1.31	0.39	4.48E-02	1.35
Serum		serine	1.21	0.27	2.84E-02	1.55
Serum		lactic acid	0.77	-0.37	1.52E-02	1.82
Serum		uridine	0.73	-0.46	1.93E-02	1.72
Serum		2-hydroxyglutaric acid	0.72	-0.47	2.93E-02	1.53
Serum		isoleucine	0.71	-0.49	2.50E-02	1.60
Serum		myristic acid	0.71	-0.49	3.77E-02	1.42
Serum		lauric acid	0.68	-0.56	3.79E-02	1.42
Serum		N-acetylmannosamine	0.66	-0.59	8.04E-03	2.09
Serum		succinic acid	0.57	-0.80	1.29E-02	1.89
Serum		2-monoolein	0.57	-0.82	4.56E-02	1.34
Serum		1-monoolein	0.53	-0.91	5.61E-03	2.25
Serum		4-hydroxyphenylacetic acid	0.49	-1.03	2.46E-02	1.61
Serum		capric acid	0.42	-1.25	2.34E-03	2.63
Serum		indole-3-acetate	0.42	-1.25	2.11E-02	1.68
Serum		adipic acid	0.42	-1.27	1.46E-02	1.84
Serum		fructose	0.36	-1.46	2.90E-02	1.54
Serum		methionine sulfoxide	0.36	-1.49	1.35E-02	1.87
Serum	HFD+LCC-RF vs HFD	uric acid	1.72	0.78	4.62E-02	1.34
Serum		glycine	1.26	0.34	1.97E-02	1.71
Serum	HFD+FMD-RF vs HFD	hexitol	3.67	1.88	1.99E-04	3.70
Serum		conduritol-beta-expoxide	3.48	1.80	5.43E-04	3.26
Serum		citric acid	1.96	0.97	3.79E-03	2.42
Serum		cysteine	1.71	0.77	4.65E-02	1.33
Serum		alanine	1.58	0.66	3.46E-02	1.46
Serum		isocitric acid	1.53	0.62	1.02E-02	1.99
Serum		2-ketobutyric acid	0.75	-0.42	2.10E-02	1.68
Serum		oxalic acid	0.74	-0.43	4.95E-02	1.31
Serum		N-acetylmannosamine	0.67	-0.59	9.60E-03	2.02
Serum		glycolic acid	0.62	-0.68	5.74E-03	2.24
Serum		cellobiose	0.59	-0.77	4.76E-02	1.32
Serum		urocanic acid	0.53	-0.92	3.47E-02	1.46
Serum		histidine	0.45	-1.16	3.96E-02	1.40
Liver	HFD+LCC vs HFD	3-hydroxybutyric acid	3.69	1.88	2.41E-04	3.62
Liver		2-aminobutyric acid	2.83	1.50	2.89E-05	4.54
Liver		inosine 5'-monophosphate	2.41	1.27	2.47E-03	2.61
Liver		pyruvic acid	2.08	1.06	2.05E-02	1.69
Liver		adenosine	1.82	0.87	1.65E-03	2.78
Liver		n-acetyl-d-hexosamine	1.77	0.82	3.57E-02	1.45
Liver		UDP-glucuronic acid	1.77	0.82	1.87E-04	3.73
Liver		inositol-4-monophosphate	1.72	0.78	3.08E-02	1.51
Liver		adipic acid	1.60	0.68	8.21E-03	2.09
Liver		pantothenic acid	1.59	0.67	4.95E-03	2.31
Liver		phosphoethanolamine	1.58	0.66	6.89E-03	2.16
Liver		2-hydroxybutanoic acid	1.55	0.63	7.85E-03	2.11
Liver		hydroquinone	1.34	0.42	1.92E-02	1.72
Liver		indole-3-lactate	1.33	0.41	1.96E-02	1.71
Liver		saccharic acid	1.30	0.38	7.37E-03	2.13
Liver		5-methoxytryptamine	1.28	0.36	4.51E-02	1.35
Liver		cholesterol	1.26	0.33	6.18E-03	2.21
Liver		palmitic acid	1.21	0.27	1.01E-03	3.00
Liver		tyrosine	0.78	-0.36	2.08E-02	1.68

Liver		fructose	0.68	-0.55	4.22E-02	1.37
Liver		histidine	0.64	-0.64	2.36E-02	1.63
Liver		ornithine 3TMS	0.64	-0.65	3.24E-02	1.49
Liver		urea	0.60	-0.73	3.21E-04	3.49
Liver		fumaric acid	0.60	-0.74	3.67E-02	1.44
Liver		glutamic acid	0.57	-0.81	1.17E-03	2.93
Liver		xylitol	0.51	-0.97	7.41E-03	2.13
Liver		alpha-aminoadipic acid	0.51	-0.98	1.51E-02	1.82
Liver		malic acid	0.44	-1.18	4.43E-03	2.35
Liver		tartaric acid	0.36	-1.47	6.19E-03	2.21
Liver	HFD+FMD vs HFD	conduritol-beta-expoxide	11.32	3.50	3.13E-05	4.50
Liver		hexitol	8.04	3.01	6.75E-06	5.17
Liver		2-aminobutyric acid	3.15	1.66	3.92E-04	3.41
Liver		cholesterone	2.85	1.51	2.38E-02	1.62
Liver		3-hydroxybutyric acid	2.85	1.51	2.81E-04	3.55
Liver		inosine	2.57	1.36	1.90E-03	2.72
Liver		creatinine	2.48	1.31	2.20E-02	1.66
Liver		adenosine	2.40	1.26	4.99E-02	1.30
Liver		phosphoethanolamine	2.38	1.25	2.70E-03	2.57
Liver		beta-alanine	2.22	1.15	8.67E-03	2.06
Liver		myo-inositol	1.93	0.95	1.42E-03	2.85
Liver		inosine 5'-monophosphate	1.77	0.83	4.04E-02	1.39
Liver		hypoxanthine	1.73	0.79	3.69E-03	2.43
Liver		ethanolamine	1.72	0.78	3.63E-02	1.44
Liver		5-methoxytryptamine	1.70	0.77	3.24E-02	1.49
Liver		threonic acid	1.67	0.74	8.34E-03	2.08
Liver		uridine	1.61	0.69	1.37E-02	1.86
Liver		UDP-glucuronic acid	1.59	0.66	2.71E-02	1.57
Liver		cellobiose	1.56	0.64	2.91E-02	1.54
Liver		maltose 1	1.55	0.63	2.82E-02	1.55
Liver		phosphoenolpyruvate	1.54	0.63	4.91E-03	2.31
Liver		galacturonic acid	1.54	0.62	2.99E-02	1.52
Liver		serine	1.52	0.60	2.68E-02	1.57
Liver		1-monoolein	1.51	0.60	2.45E-02	1.61
Liver		arabinose	1.48	0.57	1.77E-02	1.75
Liver		ribitol	1.47	0.56	2.46E-02	1.61
Liver		2-ketobutyric acid	1.46	0.55	4.63E-02	1.33
Liver		saccharic acid	1.42	0.50	1.02E-02	1.99
Liver		heptadecanoic acid	1.33	0.41	3.12E-02	1.51
Liver		3,6-anhydro-D-galactose	1.32	0.40	2.69E-02	1.57
Liver		2-deoxytetronic acid	1.28	0.36	1.44E-02	1.84
Liver		indole-3-lactate	1.28	0.35	2.32E-02	1.64
Liver		cholesterol	1.24	0.31	4.90E-03	2.31
Liver		myristic acid	0.80	-0.32	2.93E-02	1.53
Liver		levoglucosan	0.79	-0.33	3.23E-02	1.49
Liver		tyrosine	0.78	-0.36	1.81E-02	1.74
Liver		oleamide NIST	0.71	-0.50	4.16E-02	1.38
Liver		lactic acid	0.70	-0.51	2.11E-02	1.68
Liver		arachidic acid	0.70	-0.52	4.93E-02	1.31
Liver		lauric acid	0.64	-0.64	1.76E-02	1.75
Liver		proline	0.63	-0.67	2.18E-02	1.66
Liver		histidine	0.61	-0.71	1.95E-02	1.71
Liver		urea	0.55	-0.86	2.29E-04	3.64
Liver		palmitoleic acid	0.55	-0.87	3.10E-02	1.51
Liver		N-acetylglutamate	0.53	-0.92	3.86E-02	1.41
Liver		alpha-aminoadipic acid	0.51	-0.97	2.08E-02	1.68
Liver		cysteine	0.49	-1.03	4.06E-02	1.39
Liver		glutathione	0.45	-1.14	1.60E-02	1.79
Liver		tartaric acid	0.30	-1.72	3.28E-03	2.48
Liver	HFD+LCC-RF vs HFD	adenosine	1.80	0.85	7.32E-03	2.14
Liver		octadecanol	1.70	0.76	2.48E-02	1.61
Liver		pantothenic acid	1.41	0.50	7.95E-03	2.10
Liver		fumaric acid	0.42	-1.24	2.36E-02	1.63
Liver		malic acid	0.34	-1.57	8.31E-03	2.08
Liver	HFD+FMD-RF vs HFD	conduritol-beta-expoxide	3.31	1.73	8.91E-07	6.05
Liver		hexitol	2.55	1.35	2.38E-07	6.62
Liver		pyruvic acid	2.03	1.02	4.24E-02	1.37
Liver		2-monoolein	1.98	0.98	9.36E-03	2.03
Liver		isomaltose	1.46	0.54	2.53E-02	1.60
Liver		aspartic acid	0.60	-0.74	4.59E-02	1.34
Liver		malic acid	0.49	-1.02	3.42E-02	1.47

Supplementary Table 2. Metabolites implicated in the ‘Central Core of Fasting’ in the serum and liver of mice fed SD and HFD.

		Shared Metabolites under 3 days of low-calorie intake			
		LCC vs SD		FMD vs SD	
Biological Sample	***Metabolite***	***raw.pval***	***Fold Change***	***raw.pval***	***Fold Change***
Serum	conduritol-beta-expoxide	4.59E-04	2.79	4.0E-09	27.41
	3-hydroxybutyric acid	1.59E-04	14.04	1.1E-06	14.34
	lauric acid	1.18E-02	2.65	1.3E-07	4.18
	myo-inositol	2.65E-03	2.07	3.5E-06	3.44
	2,3-dihydroxybutanoic acid NIST	7.74E-06	3.55	3.4E-04	2.69
	linolenic acid	1.03E-03	4.57	4.5E-02	2.33
	mannose	1.99E-02	1.81	3.4E-04	2.32
	itaconic acid	3.20E-06	2.60	6.6E-04	2.16
	2-aminobutyric acid	2.44E-03	2.40	5.9E-04	2.15
	2-hydroxybutanoic acid	1.58E-05	3.26	1.0E-02	1.97
	fucose	5.86E-03	1.63	4.2E-04	1.94
	indoxyl sulfate	3.76E-03	1.76	5.8E-03	1.90
	threonic acid	2.84E-03	1.49	1.4E-05	1.82
	2-deoxytetronic acid	1.40E-02	1.55	2.0E-02	1.80
	glycolic acid	3.15E-03	1.48	4.6E-05	1.62
	oleic acid	1.08E-02	1.77	4.1E-02	1.60
	nicotinamide	3.49E-02	1.52	2.6E-03	1.56
	creatinine	7.96E-03	1.75	1.3E-02	1.54
	xylose	3.30E-02	1.40	9.9E-03	1.48
	thymine	2.73E-02	1.45	3.2E-02	1.46
	oxoproline	2.79E-03	1.38	2.5E-04	1.39
	glyceric acid	1.30E-02	1.78	1.6E-02	1.36
	glycerol	6.00E-03	1.52	1.4E-02	1.33
	oleamide NIST	1.60E-02	1.47	4.1E-02	1.31
	isoleucine	4.50E-04	0.61	2.9E-02	0.76
	alanine	1.30E-03	0.60	8.6E-04	0.63
	valine	8.28E-05	0.48	6.1E-04	0.61
	threonine	8.48E-04	0.53	1.5E-03	0.61
	beta-sitosterol	2.03E-02	0.51	2.0E-02	0.53
	proline	3.97E-03	0.31	4.3E-03	0.39
	tartaric acid	5.58E-03	0.04	2.8E-03	0.04
Liver	3-hydroxybutyric acid	1.82E-06	4.86	2.8E-07	4.78
	squalene	4.20E-04	2.82	2.7E-03	3.03
	inosine 5'-monophosphate	1.68E-04	3.42	2.5E-03	2.72
	myo-inositol	2.65E-03	1.43	5.0E-06	2.60
	UDP-glucuronic acid	5.64E-03	1.57	2.1E-03	2.34
	inosine	5.07E-03	1.93	2.5E-02	2.25
	guanosine	3.60E-05	1.61	1.4E-05	2.17
	hypoxanthine	1.46E-03	1.80	1.2E-02	2.16
	xanthine	4.93E-03	1.56	2.8E-02	1.73
	phosphoethanolamine	3.38E-02	1.42	1.2E-02	1.67
	cholesterol	4.80E-04	1.51	1.2E-02	1.26
	maleimide	1.86E-02	2.11	1.4E-02	1.24
	glycine	2.80E-02	0.76	2.9E-02	0.78
	dehydroascorbic acid	1.63E-02	0.78	1.0E-02	0.77
	oxoproline	1.44E-02	0.84	4.8E-03	0.75
	urea	5.62E-04	0.60	6.7E-04	0.59
	2-hydroxyglutaric acid	4.29E-07	0.48	4.3E-05	0.59
	isothreonic acid	8.01E-03	0.75	3.9E-04	0.56
	galactose-6-phosphate	2.59E-03	0.54	4.8E-03	0.55
	glutaric acid	6.27E-04	0.61	6.2E-04	0.55
	arabitol	1.52E-05	0.44	1.7E-04	0.52
	glucose-1-phosphate	5.42E-03	0.67	4.6E-03	0.44
	tartaric acid	1.63E-03	0.32	1.8E-03	0.31
	alpha-aminoadipic acid	1.02E-03	0.31	8.8E-04	0.28
	glutathione	3.24E-04	0.35	9.6E-05	0.23
	taurine	1.24E-02	0.54	1.8E-05	0.09

		Shared Metabolites under 3 days of low-calorie intake			
		HFD+LCC vs HFD		HFD+FMD vs HFD	
**Biological Sample**	***Metabolite***	***raw.pval***	***Fold Change***	***raw.pval***	***Fold Change***
Serum	2-aminobutyric acid	4.08E-06	4.84	2.4E-04	4.83
	3-hydroxybutyric acid	6.97E-04	5.59	4.6E-03	3.06
	cysteine	2.21E-02	3.36	1.1E-02	2.53
	citrulline	4.82E-02	2.02	7.1E-03	2.49
	2-hydroxybutanoic acid	4.16E-06	3.87	1.7E-02	2.06
	phosphoenolpyruvate	4.50E-02	1.53	4.3E-02	1.77
	oleic acid	1.39E-02	1.57	3.3E-02	1.74
	linoleic acid	9.05E-03	1.46	4.3E-02	1.60
	tryptophan	3.06E-04	1.73	7.6E-03	1.57
	itaconic acid	2.27E-05	2.77	6.5E-03	1.56
	N-acetyl-D-tryptophan	3.05E-04	2.53	3.3E-02	1.52
	isoleucine	2.94E-03	0.64	2.5E-02	0.71
	1-monoolein	4.93E-02	0.71	5.6E-03	0.53
	4-hydroxyphenylacetic acid	2.18E-02	0.49	2.5E-02	0.49
	capric acid	7.52E-03	0.57	2.3E-03	0.42
	adipic acid	3.14E-02	0.55	1.5E-02	0.42
	methionine sulfoxide	3.00E-02	0.51	1.3E-02	0.36
Liver	2-aminobutyric acid	2.89E-05	2.83	3.9E-04	3.15
	3-hydroxybutyric acid	2.41E-04	3.69	2.8E-04	2.85
	adenosine	1.65E-03	1.82	5.0E-02	2.40
	phosphoethanolamine	6.89E-03	1.58	2.7E-03	2.38
	inosine 5'-monophosphate	2.47E-03	2.41	4.0E-02	1.77
	5-methoxytryptamine	4.51E-02	1.28	3.2E-02	1.70
	UDP-glucuronic acid	1.87E-04	1.77	2.7E-02	1.59
	saccharic acid	7.37E-03	1.30	1.0E-02	1.42
	indole-3-lactate	1.96E-02	1.33	2.3E-02	1.28
	cholesterol	6.18E-03	1.26	4.9E-03	1.24
	tyrosine	2.08E-02	0.78	1.8E-02	0.78
	histidine	2.36E-02	0.64	1.9E-02	0.61
	urea	3.21E-04	0.60	2.3E-04	0.55
	alpha-aminoadipic acid	1.51E-02	0.51	2.1E-02	0.51
	tartaric acid	6.19E-03	0.36	3.3E-03	0.30

Supplementary Table 3. Metabolites implicated in the 'Metabolic Memory of Fasting' in the serum and liver of mice fed SD and HFD.

		Preserved under fasting and RF conditions			
		LCC vs. SD		LCC-RF vs. SD	
Biological Sample	*Metabolite*	*raw.pval*	*FC*	*raw.pval*	*FC*
Serum	3-hydroxybutyric acid	1.59E-04	14.04	5.97E-03	4.72
Serum	2-hydroxybutanoic acid	1.58E-05	3.26	2.56E-02	2.97
Serum	2-aminobutyric acid	2.44E-03	2.40	4.68E-02	2.24
Serum	lauric acid	1.18E-02	2.65	1.50E-02	2.07
Serum	glycolic acid	3.15E-03	1.48	5.76E-03	1.68
Serum	2-hydroxyglutaric acid	9.50E-03	1.37	9.35E-03	1.66
Serum	conduritol-beta-expoxide	4.59E-04	2.79	4.48E-03	1.60
Serum	pantothenic acid	5.54E-05	2.29	4.60E-03	1.59
Serum	2-hydroxyhexanoic acid	3.91E-03	1.34	1.54E-02	1.57
Serum	tocopherol alpha-	3.23E-02	0.56	4.36E-02	1.57
Serum	xylose	3.30E-02	1.40	3.42E-02	1.54
Serum	fucose	5.86E-03	1.63	4.31E-02	1.51
Serum	oxalic acid	2.81E-03	1.45	1.65E-02	1.25
Serum	valine	8.28E-05	0.48	3.89E-02	0.74
Serum	alanine	1.30E-03	0.60	2.68E-02	0.72
Serum	methionine	7.30E-03	0.41	3.69E-02	0.49
Liver	2-aminobutyric acid	3.06E-03	2.51	1.63E-02	2.84
Liver	3-hydroxybutyric acid	1.82E-06	4.86	3.39E-02	2.25
Liver	guanosine	3.60E-05	1.61	4.09E-02	1.66
Liver	1-monostearin	7.58E-03	1.66	1.60E-02	1.53
Liver	cholesterol	4.80E-04	1.51	4.06E-02	1.28
Liver	xylose	3.92E-02	1.24	2.60E-02	1.25
Liver	2,5-dihydroxypyrazine NIST	1.77E-02	0.74	4.31E-02	0.72
Liver	pipecolinic acid	1.05E-02	0.68	3.52E-02	0.72
Liver	glucose-1-phosphate	5.42E-03	0.67	3.78E-02	0.66
Liver	glutaric acid	6.27E-04	0.61	2.10E-02	0.63
Liver	arabitol	1.52E-05	0.44	3.60E-03	0.57
Liver	glutathione	3.24E-04	0.35	1.98E-02	0.50
Liver	alpha-aminoadipic acid	1.02E-03	0.31	2.72E-02	0.41
Liver	galactose-6-phosphate	2.59E-03	0.54	1.96E-03	0.36

		Preserved under fasting and RF conditions			
		FMD vs. SD		FMD-RF vs. SD	
Biological Sample	*Metabolite*	*raw.pval*	*FC*	*raw.pval*	*FC*
Serum	conduritol-beta-expoxide	4.02E-09	27.41	2.58E-06	7.27
Serum	hexitol	1.28E-09	14.65	1.86E-02	3.13
Serum	glucose-6-phosphate	1.32E-03	0.31	1.91E-02	2.25
Serum	uric acid	4.97E-03	1.32	3.96E-02	1.34
Serum	glyceric acid	1.59E-02	1.36	3.19E-02	1.31
Liver	conduritol-beta-expoxide	7.11E-05	12.54	1.79E-04	4.22
Liver	glycyl proline	2.78E-02	1.78	1.58E-02	1.79

		Preserved under fasting and RF conditions			
		HFD+LCC vs. HFD		HFD+LCC-RF vs. HFD	
Biological Sample	*Metabolite*	*raw.pval*	*FC*	*raw.pval*	*FC*
Liver	adenosine	1.65E-03	1.82	7.32E-03	1.80
Liver	pantothenic acid	4.95E-03	1.59	7.95E-03	1.41
Liver	fumaric acid	3.67E-02	0.60	2.36E-02	0.42
Liver	malic acid	4.43E-03	0.44	8.31E-03	0.34

		Preserved under fasting and RF conditions			
		HFD+FMD vs. HFD		HFD+FMD-RF vs. HFD	
Biological Sample	*Metabolite*	*raw.pval*	*FC*	*raw.pval*	*FC*
Serum	hexitol	2.96E-03	7.79	1.99E-04	3.67
Serum	conduritol-beta-expoxide	1.08E-05	10.20	5.43E-04	3.48
Serum	cysteine	1.06E-02	2.53	4.65E-02	1.71
Liver	conduritol-beta-expoxide	3.13E-05	11.32	8.91E-07	3.31
Liver	hexitol	6.75E-06	8.04	2.38E-07	2.55

Supplementary Table 4. Pathway analysis of shared metabolites in the liver of fasted and refed mice under the indicated experimental conditions.

SD context

Shared hepatic metabolites between LCC and FMD vs SD controls (Central Core of Fasting)

	Pathway	Total	Expected	Hits	Raw p	LOG(p)	Holm adjust	FDR	Impact
UPMODULATED	Purine metabolism	66	0.482	5	5.24E-05	9.86	0.004	0.004	0.179
	Ascorbate and aldarate metabolism	10	0.073	2	2.12E-03	6.16	0.176	0.089	0.250
	Steroid biosynthesis	42	0.307	2	3.56E-02	3.34	1	0.757	0.056
	Synthesis and degradation of ketone bodies	5	0.037	1	3.60E-02	3.32	1	0.757	0.000
	Butanoate metabolism	15	0.110	1	0.105	2.26	1	1	0.000
	Pentose and glucuronate interconversions	18	0.131	1	0.124	2.09	1	1	0.125
	Sphingolipid metabolism	21	0.153	1	0.144	1.94	1	1	0.014
	Galactose metabolism	27	0.197	1	0.181	1.71	1	1	0.000
	Phosphatidylinositol signaling system	28	0.205	1	0.187	1.68	1	1	0.037
	Inositol phosphate metabolism	30	0.219	1	0.199	1.61	1	1	0.129
	Glycerophospholipid metabolism	36	0.263	1	0.234	1.45	1	1	0.024
	Amino sugar and nucleotide sugar metabolism	37	0.270	1	0.240	1.43	1	1	0.017
	Primary bile acid biosynthesis	46	0.336	1	0.290	1.24	1	1	0.033
	Steroid hormone biosynthesis	77	0.562	1	0.440	0.82	1	1	0.007
DOWNMODULATED	Glutathione metabolism	28	0.260	3	1.83E-03	6.30	0.154	0.154	0.352
	Primary bile acid biosynthesis	46	0.428	2	6.58E-02	2.72	1	1	0.045
	Taurine and hypotaurine metabolism	8	0.074	1	7.22E-02	2.63	1	1	0.429
	Arginine biosynthesis	14	0.130	1	0.123	2.10	1	1	0.000
	Butanoate metabolism	15	0.139	1	0.131	2.03	1	1	0.000
	Lysine degradation	25	0.232	1	0.210	1.56	1	1	0.141
	Galactose metabolism	27	0.251	1	0.225	1.49	1	1	0.034
	Porphyrin and chlorophyll metabolism	30	0.279	1	0.246	1.40	1	1	0.000
	Glyoxylate and dicarboxylate metabolism	32	0.297	1	0.261	1.34	1	1	0.106
	Glycine, serine and threonine metabolism	34	0.316	1	0.275	1.29	1	1	0.271
	Arachidonic acid metabolism	36	0.335	1	0.288	1.24	1	1	0.000
	Amino sugar and nucleotide sugar metabolism	37	0.344	1	0.295	1.22	1	1	0.017
	Aminoacyl-tRNA biosynthesis	48	0.446	1	0.366	1.01	1	1	0.000
	Purine metabolism	66	0.614	1	0.468	0.76	1	1	0.000

SD context

Shared hepatic metabolites between LCC and LCC-RF vs SD controls (Metabolic Memory)

	Pathway	Total	Expected	Hits	Raw p	LOG(p)	Holm adjust	FDR	Impact
UP	Synthesis and degradation of ketone bodies	5	0.017	1	1.65E-02	4.10	1	1	0
	Butanoate metabolism	15	0.050	1	4.89E-02	3.02	1	1	0
	Pentose and glucuronate interconversions	18	0.060	1	5.84E-02	2.84	1	1	0.078
	Cysteine and methionine metabolism	33	0.110	1	0.105	2.25	1	1	0.042
	Steroid biosynthesis	42	0.139	1	0.132	2.02	1	1	0.028
	Primary bile acid biosynthesis	46	0.153	1	0.144	1.94	1	1	0.033
	Purine metabolism	66	0.219	1	0.201	1.60	1	1	0
	Steroid hormone biosynthesis	77	0.256	1	0.231	1.47	1	1	0.007
DOWN	Lysine degradation	25	0.133	2	6.97E-03	4.97	0.586	0.586	0.141
	Galactose metabolism	27	0.143	1	0.135	2.00	1	1	0.034
	Glutathione metabolism	28	0.149	1	0.140	1.97	1	1	0.256
	Amino sugar and nucleotide sugar metabolism	37	0.197	1	0.181	1.71	1	1	0.017

Shared hepatic metabolites between FMD and FMD-RF vs SD controls (Metabolic Memory)

	Pathway	Total	Expected	Hits	Raw p	LOG(p)	Holm adjust	FDR	Impact
UP	N/A								
DOWN	N/A								

HFD context

Shared hepatic metabolites between HFD+LCC and HFD+FMD vs HFD controls (Central Core of Fasting)

	Pathway	Total	Expected	Hits	Raw p	LOG(p)	Holm adjust	FDR	Impact
UPMODULATED	Ascorbate and aldarate metabolism	10	0.066	2	1.74E-03	6.36	0.146	0.146	0.250
	Synthesis and degradation of ketone bodies	5	0.033	1	3.28E-02	3.42	1	1	0.000
	Purine metabolism	66	0.438	2	6.78E-02	2.69	1	1	0.126
	Butanoate metabolism	15	0.100	1	0.096	2.35	1	1	0.000
	Pentose and glucuronate interconversions	18	0.120	1	0.114	2.17	1	1	0.125
	Sphingolipid metabolism	21	0.139	1	0.131	2.03	1	1	0.014
	Cysteine and methionine metabolism	33	0.219	1	0.199	1.61	1	1	0.042
	Glycerophospholipid metabolism	36	0.239	1	0.215	1.53	1	1	0.024
	Amino sugar and nucleotide sugar metabolism	37	0.246	1	0.221	1.51	1	1	0.017
	Steroid biosynthesis	42	0.279	1	0.247	1.40	1	1	0.028
	Primary bile acid biosynthesis	46	0.305	1	0.267	1.32	1	1	0.033
	Steroid hormone biosynthesis	77	0.511	1	0.409	0.89	1	1	0.007
DOWNMODULATED	Aminoacyl-tRNA biosynthesis	48	0.159	2	9.36E-03	4.67	0.786	0.556	0.000
	Phenylalanine, tyrosine and tryptophan biosynthes	4	0.013	1	1.32E-02	4.33	1	0.556	0.500
	Ubiquinone and other terpenoid-quinone biosynth	9	0.030	1	2.96E-02	3.52	1	0.729	0.000
	Phenylalanine metabolism	12	0.040	1	3.93E-02	3.24	1	0.729	0.000
	Arginine biosynthesis	14	0.046	1	4.57E-02	3.09	1	0.729	0.000
	Histidine metabolism	16	0.053	1	5.21E-02	2.96	1	0.729	0.221
	beta-Alanine metabolism	21	0.070	1	6.79E-02	2.69	1	0.815	0.000
	Lysine degradation	25	0.083	1	8.04E-02	2.52	1	0.844	0.141
	Tyrosine metabolism	42	0.139	1	0.132	2.02	1	1	0.140
	Purine metabolism	66	0.219	1	0.201	1.60	1	1	0.000

HFD context

Shared hepatic metabolites between HFD+LCC and HFD+LCC-RF vs HFD controls (Metabolic Memory)

	Pathway	Total	Expected	Hits	Raw p	LOG(p)	Holm adjust	FDR	Impact
UP	Pantothenate and CoA biosynthesis	19	0.025	1	2.51E-02	3.69	1	1	0.007
	Purine metabolism	66	0.088	1	0.086	2.46	1	1	0.001
DOWN	Citrate cycle (TCA cycle)	20	0.027	2	1.68E-04	8.69	0.014	0.009	0.074
	Pyruvate metabolism	22	0.029	2	2.04E-04	8.50	0.017	0.009	0.031
	Arginine biosynthesis	14	0.019	1	1.85E-02	3.99	1	0.518	0.000
	Alanine, aspartate and glutamate metabolism	28	0.037	1	3.69E-02	3.30	1	0.707	0.002
	Glyoxylate and dicarboxylate metabolism	32	0.042	1	4.21E-02	3.17	1	0.707	0.000
	Tyrosine metabolism	42	0.056	1	5.50E-02	2.90	1	0.770	0.025

Shared hepatic metabolites between HFD+FMD and HFD+FMD-RF vs HFD controls (Metabolic Memory)

	Pathway	Total	Expected	Hits	Raw p	LOG(p)	Holm adjust	FDR	Impact
UP	N/A								
DOWN	N/A								

Supplementary Table 5. Composition of the SD and HFD diets

Ingredients ($g.kg^{-1}$)	SD (AIN-93G)	HFD (High fat AIN-93G)
Casein, High Nitrogen	200	213
L-cysteine	3	3.0
Soy protein isolate	-	103
Sucrose	100	-
Cornstarch	397.486	127.1
Dextrose	132	-
Maltodextrin	-	62.1
Soybean Oil	70	43.009
Hydrogenated Coconut Oil	-	294
DL-Methionine	-	3.0
t-Butyl hydroquinone	0.014	-
Cellulose	50	66.141
Mineral Mix#210025	35	-
Mineral Mix#213036	-	35
Vitamin Mix#310025	10	16.61
Calcium Phosphate, dibasic	-	25.14
Calcium Carbonate	-	4.9
Choline Bitartrate	2.5	4.0
