# Supplementary material for: Diet composition influences the metabolic benefits of short cycles of very low caloric intake
Source: Nat Commun. 2021 Nov 9;12:6463. doi: 10.1038/s41467-021-26654-5 (PMC8578605; doi:10.1038/s41467-021-26654-5)
Supplement: Supplementary file 2 — Reporting Summary [file 41467_2021_26654_MOESM2_ESM.pdf]

nature research

Corresponding author(s): Rafael de Cabo

Last updated by author(s): 2021/09/02

Reporting Summary

Nature Research wishes to improve the reproducibility of the work that we publish. This form provides structure for consistency and transparency in reporting. For further information on Nature Research policies, see our Editorial Policies and the Editorial Policy Checklist.

Statistics

For all statistical analyses, confirm that the following items are present in the figure legend, table legend, main text, or Methods section.

n/a	Confirmed	
[ ]	[x]	The exact sample size (n) for each experimental group/condition, given as a discrete number and unit of measurement
[x]	[ ]	A statement on whether measurements were taken from distinct samples or whether the same sample was measured repeatedly
[ ]	[x]	The statistical test(s) used AND whether they are one- or two-sided<br>*Only common tests should be described solely by name; describe more complex techniques in the Methods section.*
[ ]	[x]	A description of all covariates tested
[x]	[ ]	A description of any assumptions or corrections, such as tests of normality and adjustment for multiple comparisons
[ ]	[x]	A full description of the statistical parameters including central tendency (e.g. means) or other basic estimates (e.g. regression coefficient) AND variation (e.g. standard deviation) or associated estimates of uncertainty (e.g. confidence intervals)
[ ]	[x]	For null hypothesis testing, the test statistic (e.g. F, t, r) with confidence intervals, effect sizes, degrees of freedom and P value noted<br>*Give P values as exact values whenever suitable.*
[x]	[ ]	For Bayesian analysis, information on the choice of priors and Markov chain Monte Carlo settings
[x]	[ ]	For hierarchical and complex designs, identification of the appropriate level for tests and full reporting of outcomes
[x]	[ ]	Estimates of effect sizes (e.g. Cohen's d, Pearson's r), indicating how they were calculated

Our web collection on statistics for biologists contains articles on many of the points above.

Software and code

Policy information about availability of computer code

Data collection	No computer codes were used in this study.
Data analysis	Excel 2019, GraphPad Prism v. 6, MetaboAnalyst versions 3.0 and 4.0, and HOMA2 calculator were used to analyze the data presented in the study.

For manuscripts utilizing custom algorithms or software that are central to the research but not yet described in published literature, software must be made available to editors and reviewers. We strongly encourage code deposition in a community repository (e.g. GitHub). See the Nature Research guidelines for submitting code & software for further information.

Data

Policy information about availability of data

All manuscripts must include a data availability statement. This statement should provide the following information, where applicable:

- Accession codes, unique identifiers, or web links for publicly available datasets
- A list of figures that have associated raw data
- A description of any restrictions on data availability

The mass spectrometry metabolomic data generated in this study have been deposited in the Metabolomics Work Bench database under accession code PR001143 (https://doi.org/10.21228/M8ZH7D). All other processed data generated in this study are provided in the Supplementary Information/Source Data file.

Field-specific reporting

Please select the one below that is the best fit for your research. If you are not sure, read the appropriate sections before making your selection.

- [x] Life sciences
- [ ] Behavioural & social sciences
- [ ] Ecological, evolutionary & environmental sciences

For a reference copy of the document with all sections, see nature.com/documents/nr-reporting-summary-flat.pdf

Life sciences study design

All studies must disclose on these points even when the disclosure is negative.

Sample size	No statistical methods were used to predetermine sample size; however, one of our recent publications has highlighted the importance of proper sample size estimation in order to ensure adequate power to detect significant group differences or treatment effects in physiological and metabolic outcomes while controlling the type I error rate (PMID: 34407413). Using these estimates, we employed n > 7, a sample size sufficient to achieve adequate power.
Data exclusions	No data were excluded from the analysis.
Replication	Several of the experiments presented in the study have been replicated in an independent cohort of animals and are featured in a companion paper #NCOMMS-21-00232A .
Randomization	Mice were randomly divided into experimental groups and assigned to receive either a standard laboratory chow or a plant-based diet (FMD) and subjected to 4:10 feeding cycles. No significant differences in body weight were observed at baseline.
Blinding	The investigators were blinded to group allocation during testing.

Reporting for specific materials, systems and methods

We require information from authors about some types of materials, experimental systems and methods used in many studies. Here, indicate whether each material, system or method listed is relevant to your study. If you are not sure if a list item applies to your research, read the appropriate section before selecting a response.

Materials & experimental systems

n/a	Involved in the study	
☐	☒	Antibodies
☒	☐	Eukaryotic cell lines
☒	☐	Palaeontology and archaeology
☐	☒	Animals and other organisms
☒	☐	Human research participants
☒	☐	Clinical data
☒	☐	Dual use research of concern

Methods

n/a	Involved in the study	
☒	☐	ChIP-seq
☒	☐	Flow cytometry
☒	☐	MRI-based neuroimaging

Antibodies

Antibodies used	Serum insulin was measured using a mouse ultra-sensitive enzyme-linked immunosorbent assay (Catalog #90080; Crystal Chem, Downers Grove, IL); Circulating levels of 3-hydroxybutyrate (3-HB) and leptin were determined with commercially available kits according to the manufacturer's instructions [3-HB: Catalog #700190; Cayman Chemicals, Ann Arbor, MI; leptin – Catalog #EZML – 82K; Millipore, Burlington, MA).
Validation	We did not perform independent validation of these commercial kits. We and others have used these kits over several years with success.

Animals and other organisms

Policy information about studies involving animals; ARRIVE guidelines recommended for reporting animal research

Laboratory animals	C57BL/6J male mice were procured from the Jackson Laboratory (Bar Harbor, ME) at the age of 47 weeks.
Wild animals	No wild animals were used in the study.
Field-collected samples	No field collected samples were used in the study.

Ethics oversight	Animal protocols were approved by the Animal Care and Use Committee (352-TGB-2019) of the National Institute on Aging, National Institutes of Health.

Note that full information on the approval of the study protocol must also be provided in the manuscript.
